# Neural signal analysis in chronic stroke: advancing intracortical brain-computer interface design

**DOI:** 10.3389/fnhum.2025.1544397

**Published:** 2025-02-21

**Authors:** Nabila Shawki, Alessandro Napoli, Carlos E. Vargas-Irwin, Christopher K. Thompson, John P. Donoghue, Mijail D. Serruya

**Affiliations:** ^1^Raphael Center for Neurorestoration, Thomas Jefferson University, Philadelphia, PA, United States; ^2^Department of Neuroscience, Brown University, Providence, RI, United States; ^3^Robert J and Nancy D Carney Institute for Brain Science, Providence, RI, United States; ^4^Department of Health and Rehabilitation Sciences, Temple University, Philadelphia, PA, United States

**Keywords:** intracortical brain-computer interfaces, neurotechnology, motor control restoration, stroke, neuroprosthetic, neurorehabilitation, microelectrode array, neuromodulation

## Abstract

**Introduction:**

Intracortical Brain-computer interfaces (iBCIs) are a promising technology to restore function after stroke. It remains unclear whether iBCIs will be able to use the signals available in the neocortex overlying stroke affecting the underlying white matter and basal ganglia.

**Methods:**

Here, we decoded both local field potentials (LFPs) and spikes recorded from intracortical electrode arrays in a person with chronic cerebral subcortical stroke performing various tasks with his paretic hand, with and without a powered orthosis. Analysis of these neural signals provides an opportunity to explore the electrophysiological activities of a stroke affected brain and inform the design of medical devices that could restore function.

**Results:**

The frequency domain analysis showed that as the distance between an array and the stroke site increased, the low frequency power decreased, and high frequency power increased. Coordinated cross-channel firing of action potentials while attempting a motor task and cross-channel simultaneous low frequency bursts while relaxing were also observed. Using several offline analysis techniques, we propose three features for decoding motor movements in stroke-affected brains.

**Discussion:**

Despite the presence of unique activities that were not reported in previous iBCI studies with intact brain functions, it is possible to decode motor intents from the neural signals collected from a subcortical stroke-affected brain.

## 1 Introduction

Stroke, a cardiovascular disease (Sacco et al., 2013), occurs when the blood supply is blocked to a part of the brain or a rupture occurs in a cerebral blood vessel (CDC, 2024a). Stroke is the leading cause of motor disability in the world, with a global prevalence of over 101 million people in 2019 (Feigin et al., 2021), and affecting over 9.4 million adults in the United States according to NHANES 2017–2020 data (Martin et al., 2024) alone with more than 795,000 cases each year (Feigin et al., 2022; CDC, 2024b). Between 60%–80% of stroke survivors suffer from permanent motor disabilities in their upper extremities (UE) or lower extremities on one or both sides of their body (Kwakkel et al., 2003; Gandhi et al., 2020). Despite rehabilitation efforts, 50%–75% of survivors continue to experience long-term motor deficit in their upper extremities that hinder their ability to complete activities of daily living (ADLs) and reduce their quality of life (Lai et al., 2002).

Intracortical brain computer interface (iBCI) technology has been proven successful in restoring various functionalities including motor and speech in persons with spinal cord injury (SCI), amyotrophic lateral sclerosis (ALS), and brainstem stroke (Hochberg et al., 2006; Donoghue et al., 2007; Collinger et al., 2013; Bacher et al., 2015; Bouton et al., 2016; Ajiboye et al., 2017; Simeral et al., 2021; Moses et al., 2021; Willett et al., 2021; Wandelt et al., 2022; Willett et al., 2023; Costello et al., 2024). In stroke patients, various non-invasive electroencephalography (EEG), and invasive electrocorticography (ECoG) techniques have been investigated to promote motor control rehabilitation (Yanagisawa et al., 2011; Gharabaghi et al., 2014; Spüler et al., 2014b,a). Despite the success of iBCIs in persons with brainstem stroke, they have not been commonly evaluated in persons with stroke above the brainstem because the risk/benefit proposition for them is distinct—for example, provision of cursor control is not as significant for a person who retains an intact hemi-body, and there is more motor function at risk of harm due to implantation). Furthermore, as cortical and subcortical strokes adversely affect more corticofugal and corticopetal fibers than brainstem strokes, it has been unknown whether the remaining cortex after subcortical stroke would produce useful motor control signals.

To the best of our knowledge, our team was the first to demonstrate that residual activity from neurons in the primary motor cortex above a subcortical chronic stroke region can be used to restore upper extremity (UE) motor control in the paretic limb using iBCI control (Serruya et al., 2022). In our proof-of-concept n-of-1 trial (Cortimo, NCT03913286), four 8 × 8 microelectrode arrays (MEAs) were implanted in the ipsilesional primary motor cortex of a person with a chronic, subcortical stroke and the recorded neural activity was decoded in real-time, enabling the participant to voluntarily operate an UE orthosis to reach, grasp, and release objects. The participant in the Cortimo trial served as his own control, as the powered orthosis operation could be triggered by surface electromyography or by the iBCI, and the performance of each was compared. Over the course of the 3-month trial, iBCI control permitted performance of a greater number of grasping tasks and at greater speed than peripheral EMG control, as measured by Jebsen-Taylor and the Action Research Arm Test (ARAT), respectively. When attempting tasks using EMG-based control, there was more resistance to orthosis motion due to abnormal muscle synergy, which was observed as forceful aberrant co-contraction of muscles across several joints. Supplementary Figure S1 shows the hand kinematics, electromyography (EMG), and associated neural data from an iBCI-controlled performance of the ARAT.

The primary motivation for using intracortical arrays was the evidence from previous human clinical trials demonstrating that trial participants were able to rapidly acquire direct voluntary control over outputs (e.g., cursors, FES) derived from the ensemble activity of multiple single neurons that can only be recorded with penetrating arrays (Hochberg et al., 2006; Ajiboye et al., 2017). Findings from the Cortimo trial showed that high gamma band local field potential neural features were necessary for behaviorally useful real-time decoding (Serruya et al., 2022). The skull is known to act as a low-pass filter, and hence this type of decoding would not be expected to be possible from scalp EEG. Scalp EEG has never been shown to provide the speed, effortlessness, and dimensionality of decoded signals as intracranial signals (Tam et al., 2019). While previous studies with ECoG have demonstrated successful motor intention decoding and wrist trajectory predictions, the ECoG grids covered intact motor-related brain areas and/or intact sensorimotor systems (Spüler et al., 2014a,b). The results in Spüler et al. (2014a) show that decoding performance was subpar for participants with more damaged motor systems using ECoG arrays. With microelectrode arrays, it is possible to target the stroke-affected region specifically and extract residual activity to drive an external orthosis, as demonstrated in the Cortimo trial.

In this paper, we present some analyses we conducted on the Cortimo trial data and discuss potential considerations to make when designing iBCIs intended to restore movement in people with hemiparesis induced by subcortical stroke. Our experience in the Cortimo trial suggests that the stroke-affected brain poses unique challenges for iBCI decoding when compared to an intact, healthy brain. First, successful iBCI decoding relies on a person to consistently generate similar ensemble activity patterns with similar timing across repeated attempts to imagine a particular movement; however a person with cerebral stroke is likely to have delayed or variable processing speed, so may be unable to generate neural activity patterns of intended movement with consistent timing relative to presented cues. Second, unlike a person who has completely lost the ability to control a limb (e.g., those with dense paresis), one with stroke typically exhibits a gradient of weakness and impairment in the limb, with some movements relatively preserved, and others significantly impaired. Hence, the distinctions between “watch, imagine, attempt" (Vargas-Irwin et al., 2018) are not as evident in persons with stroke as they are in people with complete motor disconnection. Third, in a person with stroke, aberrant motor patterns (such as agonist-antagonist co-contraction or cross-joint flexor synergies) could give rise to spastic opposition to motorized effectors, resulting in sensory feedback that reverberates back to sensorimotor cortices, confounding the decoding of their motor intent.

The purpose of this manuscript is to summarize features of neural activity that we observed and infer to be due to the subcortical stroke. Our intent is to bring to light challenges we experienced while decoding neural signals seemingly unique to a stroke brain and different from those reported in other iBCI trials. We will present a subset of data collected during the Cortimo trial and some offline analyses we conducted. We will also describe decoding strategies we developed that may inform development of iBCI devices intended to restore functional movement in people with post-stroke paresis.

In this work, we will describe electrophysiological observations of three study sessions in which our participant attempted tasks with his paretic arm while neural data was recorded from four implanted arrays, enabling us to observe neural activity at different distances from the stroke site in response to movements of his paretic hand, wrist, and entire limb, respectively. These sessions occurred during the last week of the trial on two different days: the first session consisted of a closed-loop session in which the participant used iBCI control of an UE orthosis for reaching, grasping, and releasing objects; the second consisted of an open-loop iBCI session in which he performed isometric extension and flexion of his paretic hand; and the third consisted of an open-loop session in which he conducted a planar reaching task using his paretic arm. Novel offline analysis was performed for the three sessions, namely, for the iBCI data we investigated the advantages of statistical methods for channel selection and of incorporating additional features, including phase-amplitude coupling (PAC), on motor intent decoding. For the other two data sessions, we performed offline neural population analysis to unravel new potential stroke-related neural properties.

Overall, the data collected from these sessions revealed decreased presence of gamma power, elevated delta power and frequent coordinated cross-channel firing of action potentials, which varied with respect to the distance from the stroke region, when compared to intracortical data from healthy brains. The bursting activity has not been reported in other iBCI trials, and we hypothesize that this could be the result of aberrant post-stroke physiology.

## 2 Materials and methods

### 2.1 Background—Cortimo n-of-1 trial

Details about the Cortimo n-of-1 trial, the investigational device, participant, decoders used, and prior data analyses can be found in our previous paper (Serruya et al., 2022). In brief, the participant in the trial had suffered a right hemispheric subcortical stroke that caused chronic left hemiparesis and transient aphasia. We demonstrated in the trial that residual neural activity recorded by the implanted arrays could be decoded and used to control an upper extremity powered orthosis.

#### 2.1.1 Description of the Cortimo system

**Figure 1:**
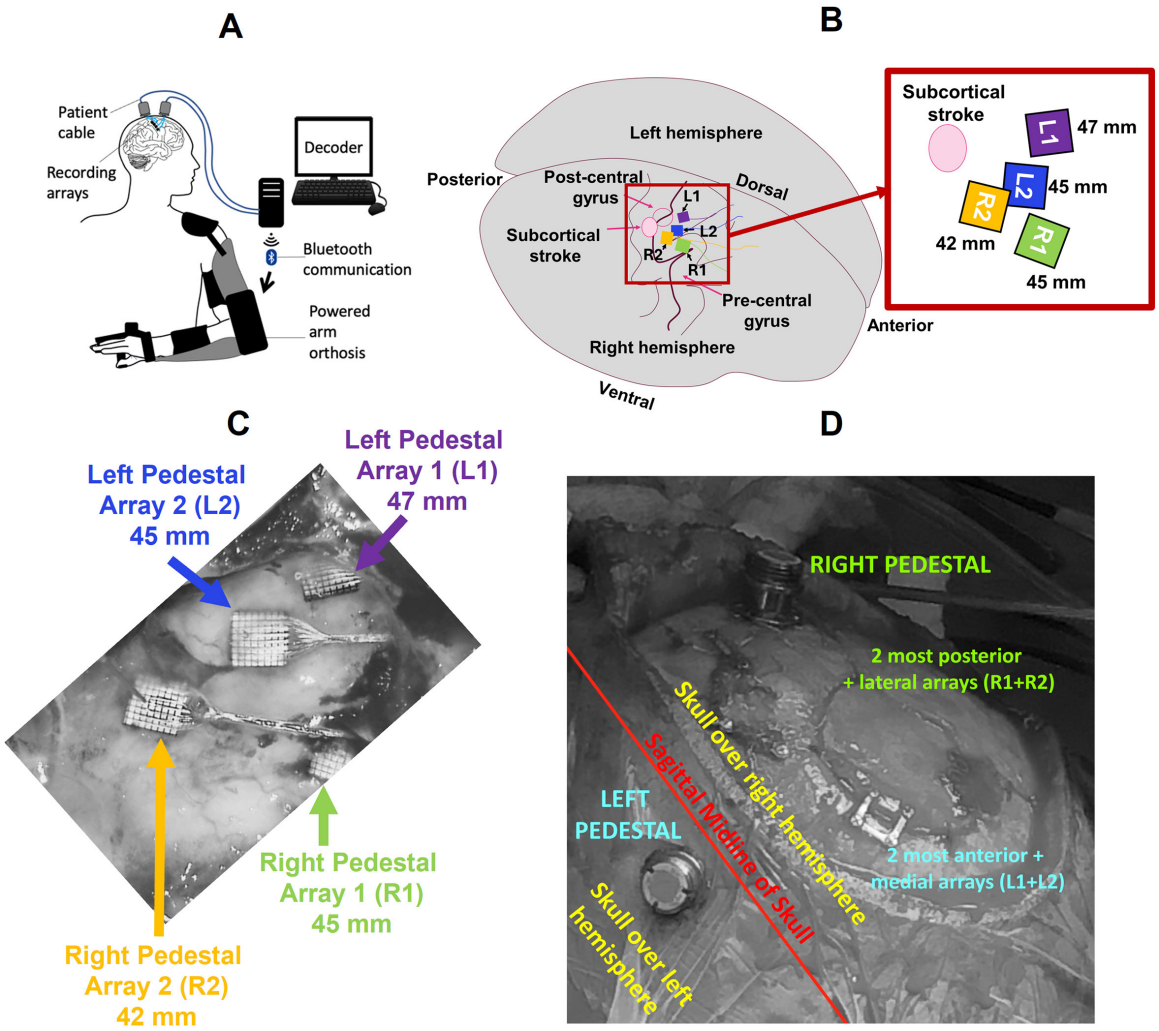
**(A)** The Cortimo System consists of recording arrays, relevant hardware, software, and a powered orthosis (image taken from Serruya et al., 2022). **(B)** Representative location of the arrays in the primary motor cortex, pre-central gyrus (not to scale). Inset: Magnified view of the arrays and their respective linear distances from the stroke site. Arrays L1 and L2 were connected to the left pedestal (LP), R1 and R2 to the right pedestal (RP), which were in turn secured to the skull over left and right hemispheres of the brain respectively. **(C)** Intraoperative view of arrays during explanation (image taken from Wu et al., 2021). **(D)** Location of pedestals on the skull with respect to the sagittal midline.

The investigational device of our trial, the Cortimo system (shown in Figure 1A), comprised the following components: (1) four implanted 64-channel arrays (NeuroPort, Blackrock Neurotech, Salt Lake City, UT) that were attached to two skull-mounted connector pedestals (two arrays per pedestal), (2) custom iBCI software, and (3) an UE powered orthosis. Two pairs of arrays were implanted in the primary motor cortex precentral knob area, and each pair was connected to a single percutaneous titanium connector pedestal (Multiport) secured to the skull. The pedestals transmitted recorded neural signals to external amplifiers and computers via a cable connection. The Cortimo software, which our team developed in MATLAB (Mathworks, Natick, MA), received the discriminated single and multi-unit signals and local field potentials (LFPs), decoded them with customized algorithms, and generated output signals to control (via Bluetooth) the motors of a commercially available powered UE orthosis (MyoPro, Myomo, Inc., Cambridge, MA). We incorporated the MyoPro, an FDA-cleared powered UE orthosis intended to facilitate movement for stroke patients, to demonstrate that neural signals could be used to restore voluntary opening and closing of the hand.

The arrays were implanted in the right hemisphere above the subcortical stroke site, and the pedestals were secured on either side of the sagittal midline of the skull. For purposes of analysis, we labeled the pedestals as Left Pedestal (LP) and Right Pedestal (RP), and their associated array pairs L1 + L2 and R1 + R2 respectively. Figure 1B shows the locations of the implanted arrays along the pre-central gyrus, the location of subcortical stroke, and the linear distances of each array from the centroid of the stroke. Figures 1C, D display the intraoperative views of the arrays and pedestal locations respectively. It is important to note that due to a malfunction during the surgical procedure one array (R1) was not properly inserted (Wu et al., 2021). Consequently, the single-unit and multi-unit spike activity that it collected was sparse and unreliable for analysis. Thus, although we analyzed LFPs from all four arrays, spike data was extracted from all but R1.

#### 2.1.2 Brief description of the full Cortimo dataset

Study visits for neural recording were scheduled for five days each week of the 3-month long trial, and multiple recording sessions could occur within a single visit. In this work, we define a recording session (“session") as one continuous period of neural recording. The complete Cortimo dataset contained ~500 recording sessions overall. In each session, raw neural data in the form of voltage time series were recorded and stored from the implanted arrays using the NeuroPort (BlackRock Neurotech, Salt Lake City, UT). The raw neural data was amplified, filtered using a first-order 0.3 Hz high-pass filter and third-order 7,500 Hz low-pass filter, and digitized at a 30 kHz sampling rate. Single unit activity and local field potentials (LFPs) were collected using this data at run-time using the Blackrock Central Software Suite (Blackrock Neurotech). We used the auto thresholding technique with a –4.5 × RMS threshold multiplier that detected a spike event if the signal energy in the 1–5 kHz range exceeded the selected threshold multiplier (Blackrock Neurotech). Along with the neural data received at 30 kHz sampling rate, The LFPs were also bandpass filtered between 0.5 and 2.5 kHz and stored at 10 kSamples/s using the Central Software. For most sessions, neural data was collected from only one pedestal at a time (i.e., from either arrays L1 + L2 or arrays R1 + R2). However, for a few sessions, the data was collected from both pedestals (all arrays). In addition to neural data, kinematics and EMG signals were collected and recorded during sessions in binary format using the Cortimo software. Elbow joint angles and hand aperture were measured by the MyoPro servomotors at 20 samples/s and root mean square (RMS) EMG envelopes of elbow and hand extensor and flexor muscles were measured by the MyoPro EMG sensors at 20 Samples/s.

In some sessions, the participant underwent isometric arm strength testing and center-out reaching experiments using the Kinarm Exoskeleton Lab system (BKIN Technologies, Kingston, ON, Canada) without using iBCI control. During these sessions, neural data was collected from the arrays along with data collected from high density surface EMG (HD-sEMG) sensor banks (OT Bioelettronica, SRL, Torino, Italy) placed along the participant's paretic arm.

### 2.2 Description of selected sessions and methodology of offline analyses

In this work, we present data and offline analysis results from these selected sessions. The goal is twofold: (1) to present a deeper investigation into innovative iBCI features and decoders that may optimize iBCI performance for the stroke population; (2) to describe our participant's paretic arm function along with neural data from all four implanted arrays. To achieve the former goal, we used data from a single recording session in which the participant used closed-loop iBCI control to trigger opening and closing of his paretic hand by the MyoPro during the Action Research Arm Task (ARAT). To achieve the latter goal, we used data from two different sessions on the same day without the use of iBCI control or the MyoPro: one in which the participant performed an isometric wrist flexion and extension task with his paretic arm and another one in which he performed various planar center-out tasks using the Kinarm Exoskeleton. Table 1 summarizes these three sessions and the types of analyses we performed.

**Table 1:** The neural recording sessions considered in this analysis.

**Activity**	**Outcome assessed**	**Duration of recording session**	**Data collected**	**Data analyzed**	**Analyses conducted**
iBCI controlled Action Research Arm Test (ARAT)	Motor function of the hand	32 min	Neural data, Muscle activity (EMG), Kinematics	LFP and spikes, Kinematics	Isolation of Oscillatory Activity, Power Spectral Density (PSD) Computation, Channel selection using spikes, Phase Amplitude Coupling (PAC)
Isometric Wrist Flexion and Extension	Muscle activity in the forearm	68 min	Neural Data, High-Density Surface EMG (HD-sEMG)	LFP and spikes, HD-sEMG	Isolation of Oscillatory Activity, PSD Computation
KinArm Visually- Guided Reaching Task	Movement of the upper extremity	35 min	Neural Data, Kinarm Data	LFP and spikes, Kinarm Data	Channel selection using spikes, PSD Computation, Population Decoding

### 2.3 Analysis of data acquired during the Action Research Arm Test

#### 2.3.1 Task description

The ARAT is a validated outcome measure of UE function that involves a series of naturalistic unimanual and bimanual tasks, such as picking up cubes of various sizes (Yozbatiran et al., 2008). In the Cortimo ARAT session, the participant performed this task using iBCI control of the MyoPro to open and close his paretic hand. He was not given visual or auditory cues to imagine or attempt a particular action but was simply given instructions and encouragement by the study occupational therapist. He performed the ARAT naturalistically at his own pace and was permitted to adjust his posture and position independent of the iBCI system to complete the tasks. The data collected during the ARAT session included hand kinematics, EMG of UE muscles, and neural data as shown in Supplementary Figures S1A–D.

#### 2.3.2 Real-time decoder description

Details of the real-time decoder that we used are described in our previous paper (Serruya et al., 2022). Briefly, we evaluated a variety of standard continuous and discrete decoders, applying them to inputs of either single- or multi-unit spike counts in 100 ms bins, or high gamma power per electrode, but the participant was unable to achieve reliable voluntary control over any of them. We investigated the signals and found they had unusual patterns, likely due to nearby white and gray matter damage. Standard decoders rely on a person mentally imagining the cued movement at a consistent timing relative to the cue: our participant, however, exhibited delayed and varying processing speed (evident even in his unimpaired hand), such that he did not imagine or attempt certain movements in a consistent manner, or generate consistent neural activity patterns. Because the standard decoders based on spike rates were not effective, we had to investigate other approaches, and ultimately developed a training-less mapping method that utilized the LFPs with a sliding-window approach. This technique triggered a closing movement of the hand using mean spectral power calculated from the LFPs, and triggered opening when LFPs corresponding to the state of “relax(ing) the hand" were detected. We calculated mean spectral power values by averaging the power in the 100–500 Hz band with 50 Hz increments over the eight most neuromodulated channels (NCs) from the L1 and L2 arrays (left pedestal, LP). The NCs were selected using the Blackrock Central Software Suite (Blackrock Neurotech) from 1-min of data recorded at the start of the iBCI session. The software identified NCs by calculating the modulation index which is the variance of firing rate changes between 0 to 50 Hz in 10 bins with 50 ms of data each. No channels from R1 and R2 (right pedestal, RP) were used. The power was calculated using 1-s sliding windows with 50% overlap from each channel in 100–500 Hz band with 50 Hz increments and then averaging across all channels and frequency bands. Supplementary Figure S1E shows the features as Power Spectral Density (PSD) averaged across the channels. We selected thresholds such that if the mean spectral power exceeded 10 V^2^/Hz, the MyoPro would close the hand, and if the signal returned to a baseline range (0.5 and 3 V^2^/Hz), it would open the hand. This training-less approach was applied in 15 sessions collected across 4 days and was found to be successful. In this manuscript, we present an analysis of a session when the participant was first learning to use the iBCI control (derived from recordings of both pedestals) to attempt portions of the ARAT.

#### 2.3.3 Frequency domain analyses

To observe the characteristics of neural data with respect to distance from the stroke region, we conducted a periodogram analysis and an Irregular Resampling Auto-Spectral Analysis (IRASA) (Wen and Liu, 2016). The periodogram enabled us to identify the activity in the frequency domain corresponding to the hand motion. Using IRASA, the oscillatory component was separated from the full spectrum to identify the active frequency bands in the data.

##### 2.3.3.1 Periodogram

To calculate the periodogram for the ARAT session, the LFP signals from all channels were first band-pass filtered from 0.5 to 1kHz using an 8th-order Butterworth filter. Then, the 60 Hz line frequency was eliminated using a band-stop filter. Then, the PSD values were calculated using Welch's method in the 0.5–100 Hz band with 0.5 Hz increment with a 1-s window and an 80% overlap to produce a feature set every 0.2 s to be used in a decoder (Shawki et al., 2023).

##### 2.3.3.2 IRASA—Separation of the oscillatory activity from the full power spectrum

Electrophysiological signals' PSD consists of two components: an aperiodic (fractal) component that follows the power law *P* ∝ 1/*f*^β^ (where *P* is power, *f* is frequency, and β is the aperiodic exponent) and a periodic (oscillatory) component containing characteristic frequency band activities that appear as spectral peaks. Previous studies showed that the aperiodic activity can change due to age and disease affecting the balance of excitatory and inhibitory synaptic currents that can be mistaken for oscillatory peaks (Donoghue et al., 2020). In stroke-affected brains, a steepened aperiodic component, reduced beta power, and slowed alpha bands were found by performing the separation of aperiodic and periodic components (Johnston et al., 2023). Moreover, using the same technique, it was found that the canonical frequency bands that are targeted for motor recovery differ in participants with disease (Stolk et al., 2019). Therefore, we hypothesized that by decomposing the total PSD into aperiodic and oscillatory components and subsequently isolating the oscillatory components, we could identify distinctive frequency bands associated with motor recovery. Investigating these relevant frequency bands would be essential to building efficient decoders for a stroke-affected brain.

To isolate the oscillatory components from the full power spectrum, we applied IRASA to the data collected from each array using the FieldTrip Toolbox (Donders Institute for Brain, Cognition and Behaviour, Radboud University, the Netherlands) implemented in MATLAB (Oostenveld et al., 2011). The IRASA algorithm separates the aperiodic component from the PSD by irregularly resampling the timeseries, against which the aperiodic component is robust, while the oscillatory component becomes distorted. Before applying IRASA to our data, the 0.5–1,000 Hz band-pass filtered signals from each channel were *z*-scored using a 30-s window. Each channel's data was divided into 4-s segments with a 75% overlap to capture two cycles of the 0.5 Hz component along with a 1-s increment. To determine the aperiodic component, the algorithm up-sampled the timeseries using 17 resampling factors ranging from 1.1 to 1.9 with 0.05 increments and down-sampled the timeseries using the inverse of these resampling factors. For each of the up-sampling and down-sampling pairs, the geometric mean of the PSD was computed, and the median of all pairs was calculated—which was the aperiodic component of power spectra of the given timeseries. The oscillatory component was then calculated by subtracting the aperiodic component from the total PSD.

For the ARAT session, 32 min of consecutive LFP data was considered from all the channels of each array. The LFP data was first band-pass filtered from 0.5 to 1kHz using an 8th-order Butterworth filter. Then, the 60 Hz line frequency and its harmonics were eliminated using band-stop filters. After *z*-scoring the data using 30 s of window, IRASA was applied to 0.5–30 Hz band for this session with a 4-s window and 1-s increment so that two cycles of 0.5Hz component were included.

#### 2.3.4 Offline responsive channel characterization technique

The novel offline analyses presented in this paper include selecting channels for feature extraction using recorded spike, and subsequently extracting features from the LFPs. We identified channels offline by applying a responsive channel (RC) characterization technique on the signals that were classified as spikes (which may have reflected single-unit or multi-unit activity recorded at that channel) (Hosman et al., 2021). This technique compared the spiking activities of each channel during the hand closing and opening events and labeled a channel as responsive if the difference between the two was statistically significant (Kruskal Wallis [KW], *p* < 0.01) (Hosman et al., 2021).

Hand kinematic data was recorded as grip aperture (percentage of the paretic hand's range of motion), where the hand was considered fully open at 100% and fully closed at 0%. This data was later analyzed to identify opening and closing events. A thresholding technique was applied to find valid closing and opening events. A closing event was defined as the occurrence of the hand initiating closing from an 80% or more open position and reaching the fully closed position (0%). If the hand motion stopped and resumed during a closing action, the latter closing action that reached close (or at least 30% closed position) was considered the closing event. Likewise, an opening event was considered if the opening was initiated at 30% or less closed position and reaching an open position (at least 80%). If the hand motion stopped and resumed during an opening motion, the latter opening movement that reached open (or at least 80% open) position was considered as the opening event.

**Figure 2:**
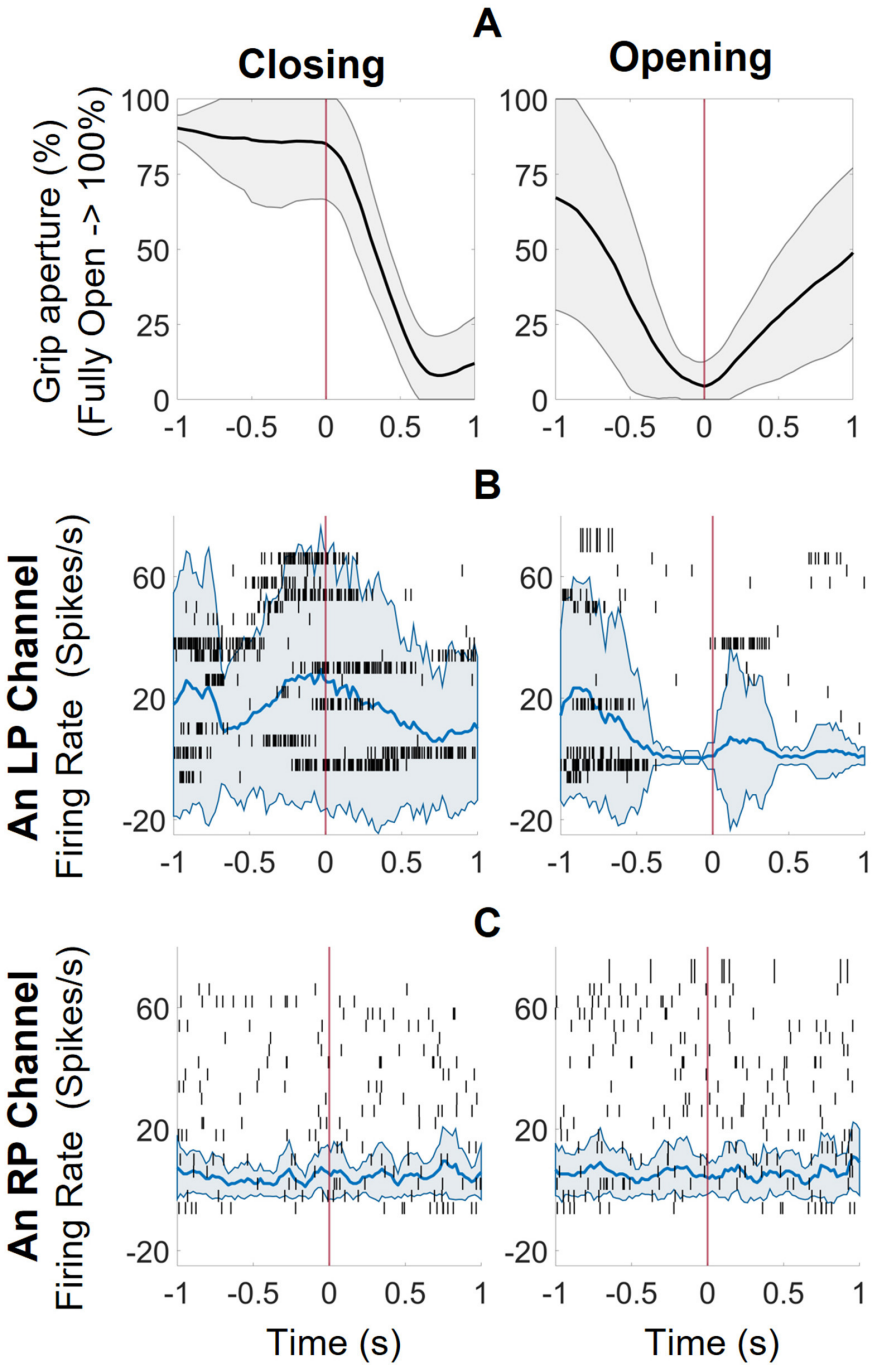
The responsive channel characterization technique. **(A)** the average trajectories of 19 closing and 21 opening events, aligned at the start of the event with spikes from one second before and after **(B)** The raster plot of a representative channel from the left pedestal (LP, L1 + L2) with mean and standard deviation of firing rates for closing and opening events (calculated with 100 ms windows, 20ms increments). There was increased activity before initiation of a closing event and low to no activity at initiation of open event (consistent with participant instructed to relax when he wanted to open his hands). **(C)** The raster plot of a representative channel from the right pedestal (RP, only R2 since R1 was incorrectly implanted) with mean and standard deviation of firing rates. There is no activity pattern associated with the closing or opening event.

To identify the RCs in arrays L1, L2 and R2 (R1 data was not used for reasons described previously), we selected 400 s of kinematic and corresponding neural data, which contained 19 closing events and 21 opening events. This region was chosen because it contained a significant number of closing events with a full range of motion (fully open at 100% to fully closed at 0%). The average trajectories of these events are shown in Figure 2A. Because the closing events were not externally cued, we assumed that 1 s of data preceding the event would provide enough data to capture the motor planning. Event-related spike data was windowed using 1 s pre- and 1 s post- event detection; such data windows were defined as the event-related spike activity. The firing rates calculated in each 100-ms window during the closing and opening events were compared using KW analysis. The raster plots of two representative channels from LP and RP corresponding to closing and opening events along with mean and standard deviation of the firing rates are shown (Figures 2B, C).

Using KW analysis, it was observed that in the 240-ms window before an event initiated, firing rates from 57% (73 out of 128) left pedestal channels were significantly different (KW, *p* < 0.01) between the closing and opening events, and hence were denoted as RCs. For the right pedestal, only 1 of the 64 channels (from R2) had firing rates statistically different between closing and opening events, in the windows 0.5 and 0 s before an event initiated. To compare the data from both pedestals, we selected features of the channel at 0.16 s before the event started for further analysis.

None of the channels used in the real-time ARAT task were found to be responsive using the offline statistical technique. This discrepancy likely arose because the NCs used for the ARAT online decoder (using LFPs) were selected using the modulation indices (using spikes) derived from the Central software with 1 min of data collected prior to the start of the ARAT online-decoding session as described in Section 2.3.2. However, the offline derived RCs were computed using 400 s of ARAT data (online decoder) and the KW analysis. The feature extracted from the LFPs of original NCs were stable enough to drive the MyoPro device in real time (online decoder) as shown in Supplementary Figure S1E even though the spikes were not found to be significantly different using the offline data and KW analysis.

#### 2.3.5 Offline feature extraction methods

To extract features corresponding to the closing events, the LFP data from the RCs was first band-pass filtered from 0.5 to 1kHz using an 8th-order Butterworth filter and the 60 Hz line frequency component was removed using a band-stop filter. We investigated the use of phase-amplitude coupling (PAC) as an input feature for motor intent decoding and to see if it might provide insight into the dynamic behavior of the neuronal organization. We calculated the PAC between the phases of the low-frequency band and the amplitude of the high-frequency. We quantified the PAC using a modulation index (MI) method, which uses the analytic signal to quantify the PAC between the slower and faster neuronal oscillations (Tort et al., 2010). We applied this technique to the phases of 1–4 Hz low frequency band (Ramanathan et al., 2018) and high frequency amplitudes of 70–450 Hz in 1-s windows with 80% overlap to decode an output every 0.2 s. The signals were first filtered in these frequency ranges with band-pass filters, and the appropriate phases and amplitudes were then extracted from the analytic signal generated using the Hilbert transform. The –180° to 180° phase range was separated into 20 bins, 18° apart. The high frequency bands' amplitudes were then accumulated in the corresponding binned phases, and the mean of the amplitudes in each bin was calculated. The mean amplitudes were then normalized by dividing the bin value by the overall sum, per the equation:


(1) $$\begin{array}{l} p\left(i\right)=\frac{\bar{A}\left(i\right)}{\overset{N}{\underset{k=1}{\sum }}{\bar{A}}_{k}}, \end{array}$$

where, *p*(*i*) is the normalized amplitude of the *i*^*th*^ bin, $\bar{A}\left(i\right)$ is the mean amplitude in the *i*^*th*^ bin, *N* is the total number of bins, and *k* is the index of the bins. Finally, the modulation index was calculated using the Kullback-Leibler (KL) distance ranging from 0 to 1, where 0 indicates no existing phase-amplitude coupling between the low-frequency phase and high-frequency amplitudes. In such a case, the amplitude distribution, *p*, resembles a uniform distribution. For our analysis, we computed the average PAC values corresponding to 19 closing tasks.

### 2.4 Offline analysis of data acquired during isometric flexion and extension of the paretic wrist

In this session, neural data and HD-sEMG data were recorded while the participant performed isometric contractions of his forearm muscles. The participant was seated in a chair instrumented with an isokinetic dynamometer (BioDex; Shirley, NY) and was strapped in securely to limit movement. Two 64-channel HD-sEMG sensor banks (8 mm inter-electrode distance, five columns × 13 rows, ELSCH064NM2; OT Bioelettronica; Turin, Italy) were placed one each over the wrist extensors and wrist flexors of the paretic arm. Neural data was collected from both pedestals while the participant performed several verbally cued wrist extension and flexion tasks, with cues given before every task. No real-time decoding was performed during these tasks. The HD-sEMG data was saved in proprietary OTB format (OT Biolettronica) and the neural data was recorded with the Nuroport. Furthermore, one EMG channel and a synchronization pulse train channel were shared between the two acquisition systems for high temporal resolution offline data synchronization. Th HD-sEMG data was converted to MATLAB's data format, MAT, using the OTBioLab+ software (OT Biolettronica). The HD-sEMG and neural data were synced using cross-correlation analysis in MATLAB using the common EMG channel and sync pulses. Three trials of maximum voluntary isometric wrist extension (“wrist extension max," WEM) were chosen for further analysis.

#### 2.4.1 Frequency domain analysis

##### 2.4.1.1 Periodogram

The neural data from the three WEM trials were manually annotated into three time periods based on the sEMG responses: pre-task, during-task, and post-task. Using periodogram and IRASA analyses, the neural activity occurring during the WEM task was analyzed. The LFP signals from all channels were first band-pass filtered from 0.5 to 1kHz using an 8th-order Butterworth filter. Then, the 60 Hz line frequency was eliminated using a band-stop Butterworth filter. The resulting data was *z*-scored using a 30-s sliding window. The PSD values were calculated using Welch's method using a 1-s window with an 80% overlap from 70 to 450 Hz with 0.5 Hz increment. This approach yields a new decoding output every 0.2 s.

##### 2.4.1.2 Oscillatory activity

To find the oscillatory characteristics in the pre-task, during-task, and post-task time periods, we applied the IRASA method described in Section 2.3.3. The filtered and *z*-scored LFP data from each period were analyzed across the three WEM trials using the MATLAB FieldTrip toolbox.

### 2.5 Analysis of data acquired during performance of Kinarm visually guided reaching task

Neural data and kinematics were recorded while the participant performed reaching center-out tasks with the affected and unaffected arm using a Kinarm Exoskeleton Lab. Various types of data were collected by the Kinarm, including position, angular velocity, angular acceleration, force, and torque in multiple dimensions. The sync pulses from NeuroPort were also recorded in the session for synchronizing the neural data with the recorded Kinarm data. No real-time decoding was performed during these tasks.

The Kinarm allowed free movement of the proximal and distal arm in horizontal planes, facilitating flexion and extension of the elbow and shoulder joints. The participant operated the Kinarm manipulandum during the tasks, which were externally cued by a red target displayed on a computer screen. While performing tasks with the paretic arm, the participant was permitted to use the unaffected arm for assistance.

For each trial, the participant was asked to move a cursor to reach one of eight targets that were arranged in a circle equidistant from a center position and separated by 45°. Each trial involved toggling between a center target and one of the eight targets (selected randomly) within 14 s. In the given trial time, the participant was instructed to toggle between the center and the selected directional targets as many times as possible. After a break, a new trial would begin with a different peripheral target. We selected two center-out trials for further analysis: one performed with the unimpaired arm and one with the assisted paretic arm. Because we had limited time to perform this session, the participant only performed two to four trials in each of the eight directions. Supplementary Figure S2 displays the reaching trajectories for all targets from these two trials; the movements to and from the bottom-left (BL) target for both arms are expanded in the insets.

#### 2.5.1 Frequency domain analysis

To observe the frequency domain activity of the arrays during the center-out task session, we computed the periodogram and applied the IRASA method described in Section 2.3.3. The LFP signals from all channels were first band-pass filtered from 0.5 to 1kHz using an 8th-order Butterworth filter. Then, the 60 Hz line frequency was eliminated using a band-stop Butterworth filter. The data was *z*-scored using 30 s of previous data. The PSD values were calculated using Welch's method using a 4-s window with a 75% overlap from 0.5 to 100 Hz with 0.5 Hz increment that captures a decoding output every 0.2 s. The IRASA method was applied using the same 4-s window with a 75% overlap to capture the full two-cycle of 0.5 Hz frequency component.

#### 2.5.2 Neural population decoding of Kinarm activity

To understand if the directions of the center-out task were encoded in the neural population, we performed population decoding using the spike activity. We applied a responsive channel characterization technique described in Section 2.3.4 to find the channel for feature extraction by comparing baseline and movement spikes. Due to the limited number of trials, we combined both center-to-out data for a given target, and out-back-to-center data for the target directly opposite (e.g., data collected while moving from the center to the right target was combined with that collected while moving the hand from the left target back to center). By considering both center-out and out-to-center movements in each of the eight directions, we were able to collect 24 movements for the paretic arm (three in each direction) and 26 for the unimpaired arm (three movements each, except for 4 in the left and bottom directions) The baseline spikes for each target were collected for 1 s starting from 0.25 s after reaching the previous target. The movement spikes were collected for 1 s starting from 0.25 s before and 0.75 s after the appearance of a new target. The firing rates were calculated for each 1-s spike activity with 300-ms windows and 20-ms increments. Using the Kruskal Wallis analysis (*p* < 0.01), We found 19 and 12 (of 128 left pedestal channels) responsive channels (RCs) respectively for the unimpaired and assisted paretic arm tasks.

**Figure 3:**
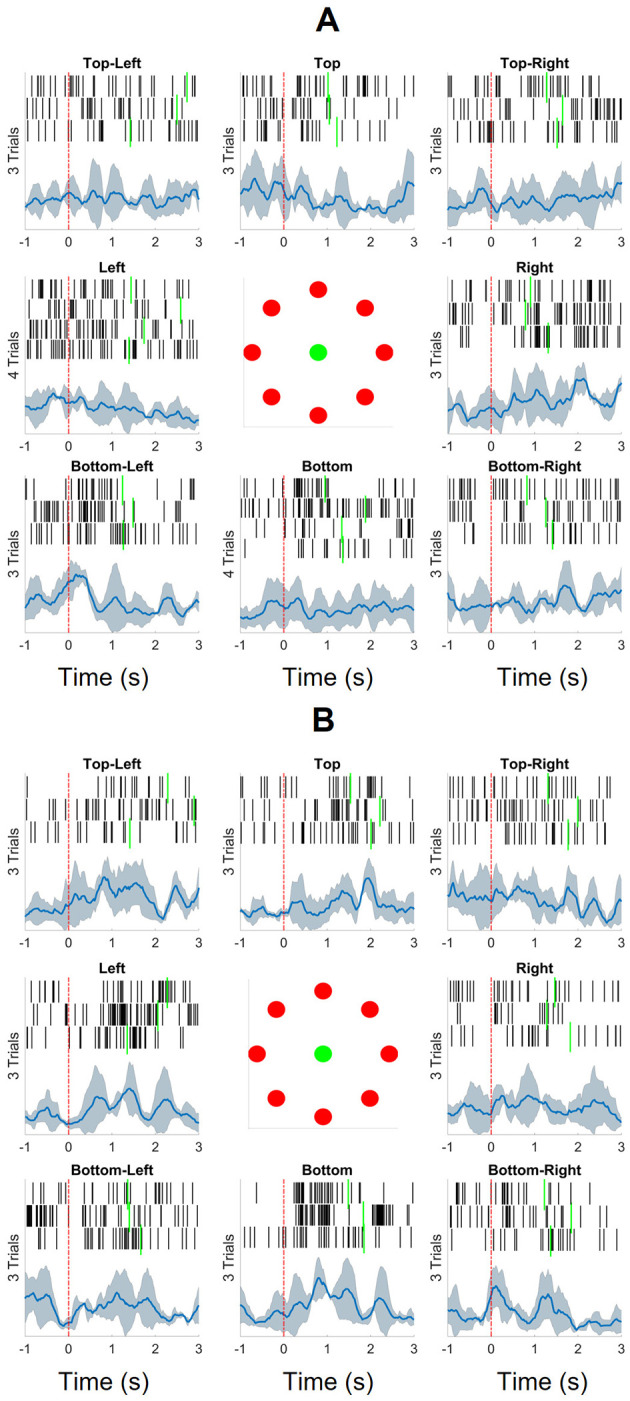
The spikes and normalized leaky integrated firing rates (NLIFRs) aligned at the time of target appearance (indicated by the red dashed line) for eight directions for both the unimpaired right and paretic left arms. The target reach times are noted with the green solid lines. The spikes were collected with a 300 ms sliding window with 20 ms increment and then the leaky integrated firing rates were calculated and normalized by the maximum firing rate within the (–1, 3.5)-s long segments. **(A)** The participant reached 26 targets in total with his unimpaired right arm. **(B)** He was able to reach 24 targets with his paretic left arm that was assisted by his unimpaired right arm.

The channels identified as responsive were used to calculate normalized leaky integrated firing rates (NLIFR) with 300-ms sliding windows with a 20-ms increment. The raster plot and the averaged NLIFR for all movements within 1 s before and 3 s after the target appeared are shown in Figure 3. For feature extraction, we chose a segment of 0.5 s before and 1 s after a new target appearance to investigate the movement planning phase. The NLIFRs were normalized by the maximum firing rate occurring within this chosen segment.

A linear model, Linear Support Vector Machine (linear SVM), and, a non-linear model, k-nearest neighbor (kNN), were chosen to classify the eight directions. Linear SVM has been widely used for motor intention decoding and wrist trajectory prediction because of its superior generalization capabilities and performance reliability in high-dimensional feature space (Yanagisawa et al., 2011; Spüler et al., 2014b; Schaeffer and Aksenova, 2018). The kNN algorithm has been utilized for offline neural signal classification and predictions, such as motor imagery and arm movement classification, in various studies (Chin et al., 2007; Ifft et al., 2013; Kayikcioglu and Aydemir, 2010; Cajigas et al., 2021) due to its simplicity and ease of implementation (Schaeffer and Aksenova, 2018).

We evaluated the neural features across four distinct experiments. First, in 50 iterations, two samples for each direction were chosen randomly for training and the rest for testing. Then, to investigate the relationship between the samples and the labels, we performed permutation testing by shuffling the labels with 500 iterations with same training and testing split. Again, to explore the importance of the responsive channels, we chose 100 sets of 20 random channels from 128 channels of L1 and L2 and ran 50 iterations on each set. Lastly, all 128 channels of L1 and L2 were tested with 50 iterations. Five metrics were chosen for comparing the performance models: average accuracy, average precision, average recall, average specificity, and average F1-score (Manning et al., 2008).

## 3 Results

### 3.1 The iBCI ARAT session

#### 3.1.1 Frequency domain analysis

**Figure 4:**
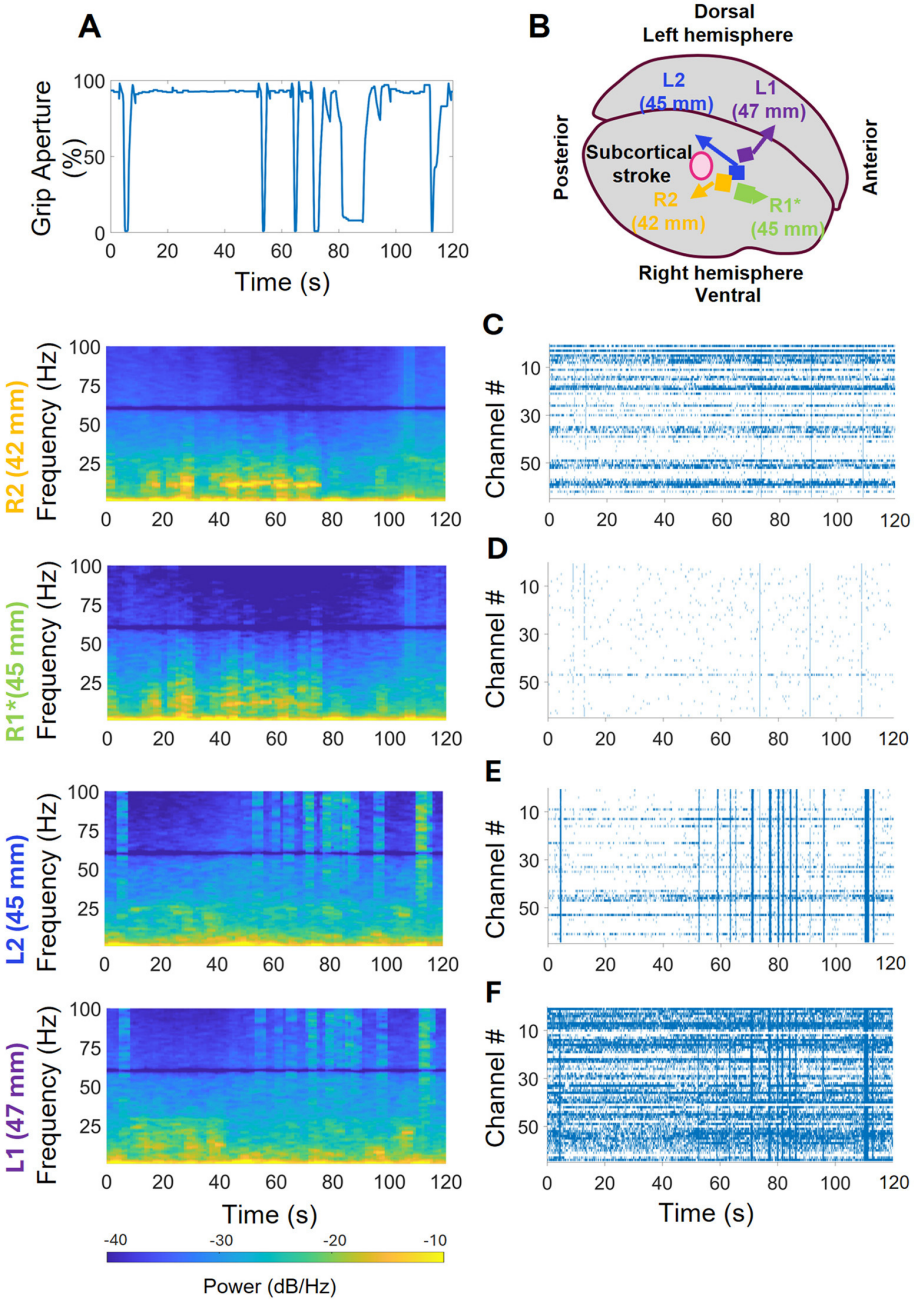
Neural activity recorded by arrays during the iBCI ARAT session. **(A)** Kinematics of hand closing events during iBCI control of orthosis (hand was fully open at 100% and fully closed at 0%). **(B)** Array positions with respect to the stroke location. **(C)** R2, closest to stroke region, did not have strong high frequency activity; but some rhythmic activity in the low frequency region when the hand remained open, and a high frequency burst. The spike raster plot shows some coordinated cross-channel firings lasting for less than a second which coincident with the hand closing events shown in **(A)**. **(D)** R1 was incorrectly implanted but shows similar high and low frequency activity as R2 in the periodogram. Despite not collecting enough spikes, it did show brief, coordinated cross-channel firings aligned with those of R1. **(E)** L2 had strong high frequency and coordinated cross-channel firings coincident with closing events, and no low frequency rhythmic activity. **(F)** L1, farthest from stroke region, showed strong high frequency activities coincident with closing activities and some low non-rhythmic frequency activities while the hand remained open. The raster plot shows activity corresponding to the closing events.

Power spectral analysis of neural activity recorded from the four intracortical arrays revealed a pattern where high frequency power was attenuated and low frequency power increased in the arrays closer to the centroid of the underlying subcortical stroke (R1 and R2), relative to the arrays further away (L1 and L2). In addition to this pattern, which was present throughout the duration of the session, superimposed patterns that fluctuated were also evident, with periodic bursts of low-frequency power seen in R1 and R2 and fluctuations in high frequency activity corresponding to hand motion seen in L1 and L2. Figure 4A displays the hand kinematics during 120 s of the session, and Figures 4C–F display the periodogram and spike raster plots for each array during that time. Figure 4B shows the array location in the precentral gyrus for reference (not to scale). Figure 4C shows the neural activity associated with R2 which is the closest to stroke region: rhythmic activity in the 7–18 Hz ranges from 5–78 s can be observed when the hand stayed open for about 50 s; this rhythmic 7–18 Hz activity was also seen in the R1, which was not fully implanted into the cortex (Figure 4D). In the R2 array, a high frequency burst is noticed at the 106th s. The spike raster plot shows a few episodes of coordinated cross-channel firing occurring around hand closing events shown in Figure 4A. The R1 array did not collect spikes which is evident in the raster plot. In the L2 array, shown in Figure 4E, no low frequency rhythmic activity was observed, but strong high frequency activity was observed corresponding to the hand closing events. The raster plot shows coordinated cross-channel firing happening at those times as well. Finally, Figure 4F shows the average frequency domain activities and spike raster plot of the L1 array, the farthest from the stroke. It demonstrates that there were strong high frequency activities and some non-rhythmic low frequency activities when the hand stayed open between 5–55 and 90–115 s. The raster plot shows more activity in L1 than in all other arrays, as well as coordinated cross-channel firing that aligned with the hand closing events and the high frequency activity in the periodogram.

#### 3.1.2 Oscillatory activity

**Figure 5:**
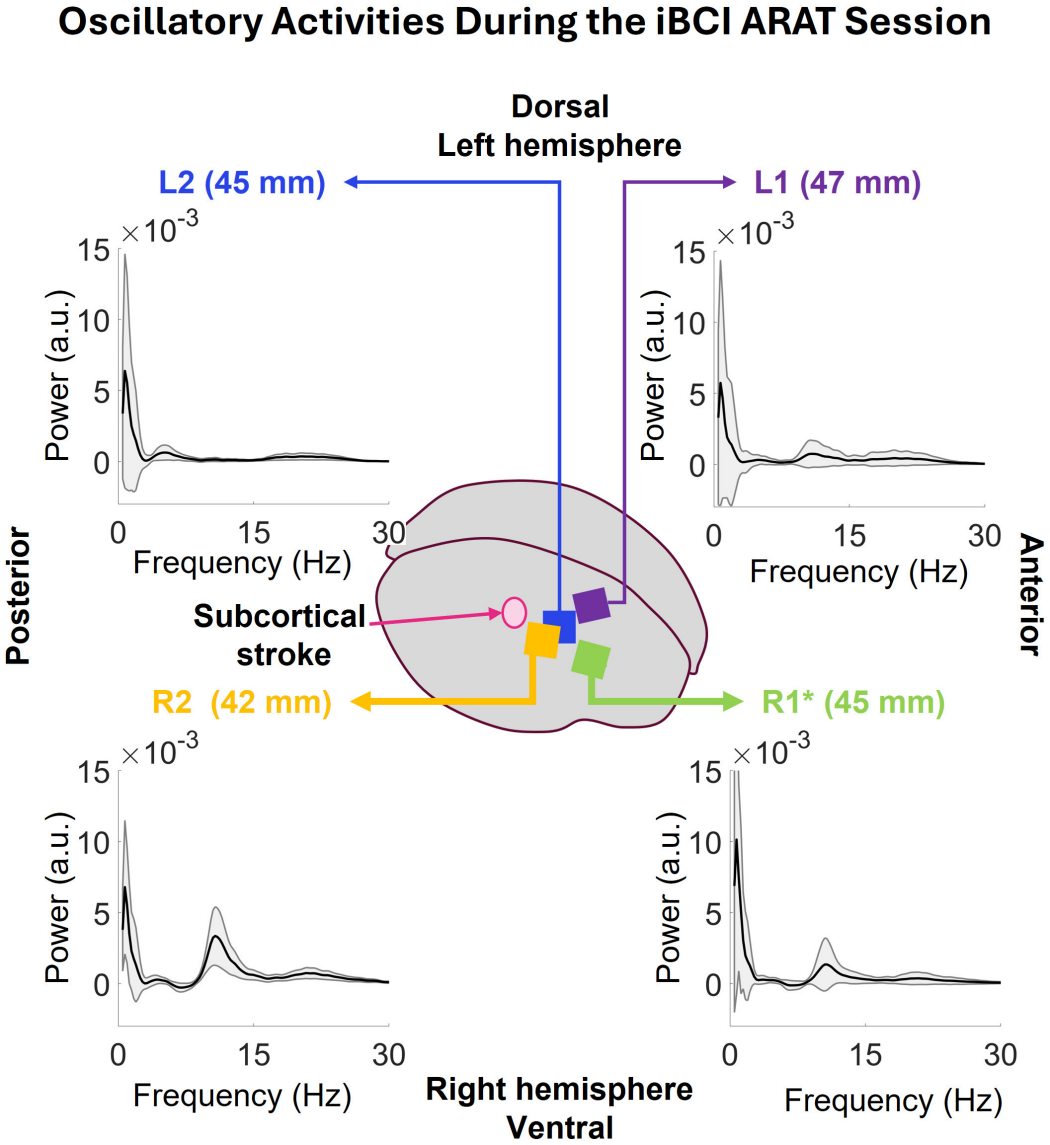
Oscillatory activity during the full iBCI ARAT session, extracted using the IRASA method for each individual array. The oscillatory activities show a peak at 0.73 Hz that is common across all arrays. in the lower-left corner, R2 is the closest array to the stroke region and displays the strongest peak at 0.73 Hz among all the correctly implanted arrays. The peak at 9–14 Hz range can be a result of the rhythmic activity in this array as shown in the periodogram data. Next, in the lower-right corner, R2's average peak at 0.73 Hz is stronger than R1, but, since it was not correctly implanted, it might be due to high impedance. The latter peak at 9–14 Hz is weaker than that of R1. In the upper-left corner, L2's peak at 0.73 Hz is slightly weaker than that of R1. It does not have any activities like R1 or R2 but has some activities in 3–9 and 16–27 Hz bands. Lastly, upper-left corner shows the oscillatory activity of L1 where the average peak at 0.73 is weaker than R1 and R2 as well. There are some activities in 8–27 Hz band.

Figure 5 displays the oscillatory activity in the 0.5–30 Hz range associated with the full iBCI ARAT session. The highest peak of average oscillatory activity for all arrays was at 0.73 Hz. They also show some activity in the 17–28 Hz range. Starting with the closest array to the stroke site, R2, on the lower left corner, the average peak at 0.73 Hz is strongest in this array at 6.79 ± 4.70 × 10^−3^ a.u. (arbitrary unit) among all the correctly implanted arrays. A narrow peak at 9–14Hz range is visible as well that can be a result of the rhythmic activity shown in PSD in Figure 4C occurring all throughout the session. In the lower-right corner of the Figure 5, one can see that R1's average peak at 0.73 Hz (10.10 ± 10.86 × 10^−3^ a.u.) is stronger than R2, but, since it was not correctly implanted, this power can be due to higher impedance. The latter peak at 9–14 Hz is weaker than that of R1. On the upper-left corner, L2's 0.73 peak has a power of 6.38 ± 8.24 × 10^−3^ a.u. along with some activity in 3–9 Hz band. Lastly, the farthest array from the stroke region, L1, shows the weakest peak at 0.73 Hz with a power of 5.72 ± 8.63 × 10^−3^ a.u. the oscillatory activity also displays activity in the 9–27 Hz range.

#### 3.1.3 Offline features

**Figure 6:**
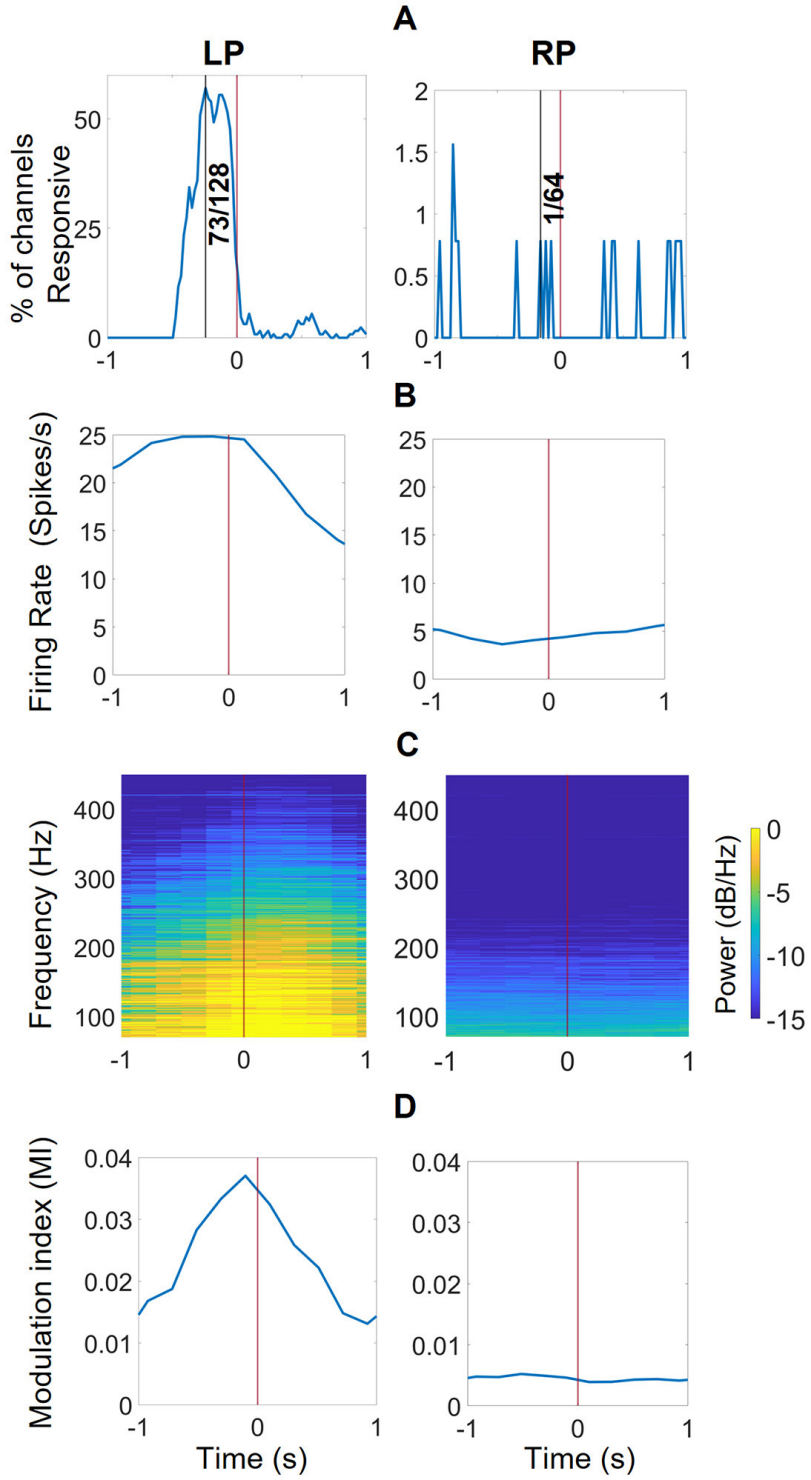
Offline features extracted from responsive channels correlated with closing events for the iBCI ARAT session. **(A)** The maximum number of responsive channels in this 2-s range at 0.24 s before the closing event. Out of 128 channels of the left pedestal (LP, L1 + L2), 57.00% or 73 channels were responsive, (i.e., the firing rates were significantly different (KW, *p* < 0.01) during closing compared to opening) were responsive. On the other hand, only 1/64 channels from the right pedestal (RP, R2) was responsive. R1 was not included in this analysis as it was not implanted correctly. **(B)** The average firing rate during closing events for all responsive channels. **(C)** the average PSD features of the responsive channels in the 70–450 Hz region with 10 Hz increment, averaged for all closing events. **(D)** the phase-amplitude coupling (PAC) features between the phase of 1–5 Hz and amplitudes of 70–450 Hz band, calculated and averaged for all closing events using modulation index (MI) method across all responsive channels.

Using the responsive channel characterization technique described in Section 2.3.4, we identified 73 responsive channels for the LP (L1 + L2) and 1 for the RP (R2) as shown in Figure 6A. Using these channels, we calculated the average firing rates, average PSD, and average MI values associated with all closing hand events. Figure 6B displays the average firing rates for LP and RP channels, calculated with a 1-s sliding window with a 200 ms increment (80% overlap). The LP channels' firing rates increased before the closing events initiated and fell once the hand started to close; the RP channel firing rates did not exhibit any pattern associated with the events. Additionally, PSDs were computed with the periodogram method on MATLAB from the identified RCs using a 1-s sliding window with an 80% overlap, 70–450 Hz band with 0.5 Hz increment. As shown in Figure 6C, in the average periodogram of the LP channels, the PSD values became stronger before the closing event initiated and weakened once the movement happened; however, R2 showed weak activity in this high frequency band, suggesting that neural activity recorded in that area of the brain was not coupled to intended hand movement. Figure 6D displays the MI, calculated using a 1-s sliding window with an 80% overlap. The average MI in LP channels increased before the closing event was initiated and decreased when movement started happening, suggesting a correlation with motion planning.

### 3.2 The isometric wrist flexion and extension task

#### 3.2.1 Frequency domain analysis

**Figure 7:**
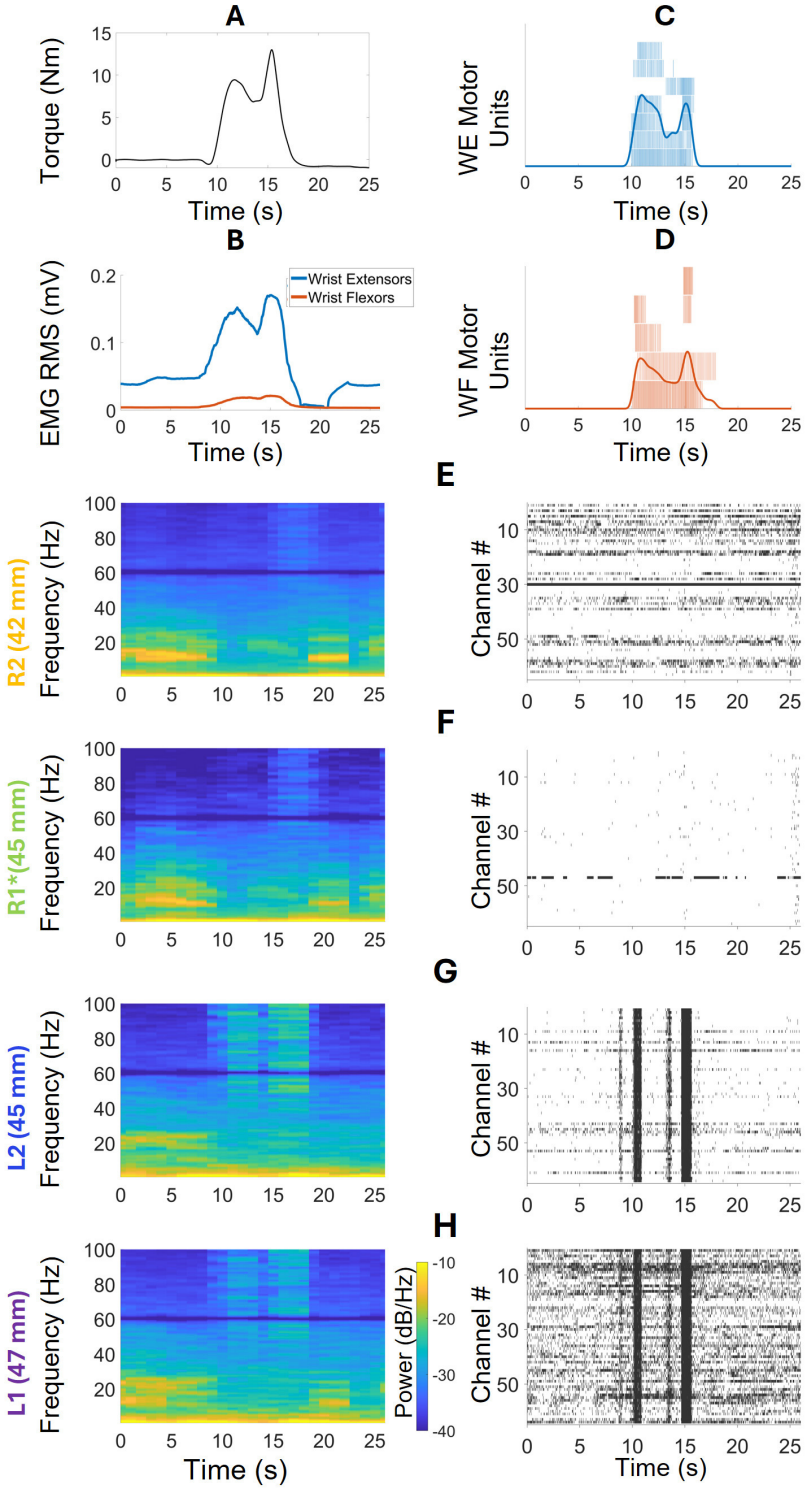
Frequency Domain Analysis and spike raster plots during a WEM task. **(A)** Wrist torque during the task. **(B)** RMS EMG envelopes of wrist extensors and flexors, indicating co-activation. **(C)** Individual and cumulative spike trains of seven motor units from the HD-sEMG data of the wrist extensors. **(D)** Individual and cumulative spike trains of five motor units extracted from the HD-sEMG data of the wrist flexors. **(E)** The periodogram and raster plot corresponding to the WEM task in R2. The periodogram shows high-frequency activity before and after the task, which disappears when the task begins at the 9th s. During the task, high-frequency activities are visible at 15–18 s. The raster plot does not show any visible patterns corresponding to the task. **(F)** R1 shows behavior similar to R2 in both the periodogram and raster plots. **(G)** L2 shows low-frequency activity only before the task starts. During the task, high-frequency activity is seen throughout the entire duration, with no visible activities after the task. The raster plot shows coordinated cross-channel firings during the task. **(H)** L1 shows low-frequency activity both before and after the task, as well as high-frequency activity during the task. The raster plot is more active for this array, also showing coordinated cross-channel firing during the task.

We chose three WEM tasks for further analysis in the open-loop iBCI isometric Wrist Extension and Flexion session. Along with the neural data, muscle activity from the wrist extensors (WE) and flexors (WF) were calculated using HD-sEMG sensors. Figure 7A displays the torque of the paretic wrist during the extension movement. Figure 7B shows the RMS EMG envelopes for the wrist flexor and extensor muscles during the task, indicating that while the wrist extensors were stronger than the flexors, there was co-activation. Figures 7C, D display the motor units and cumulative pulse trains of wrist extensors and flexors respectively, which also indicate co-activation. The periodograms and spike raster plots shown in Figures 7E–H display the neural activities in the arrays, in order of closest to farthest distance from the stroke location. In R2, Figure 7E, some activity in the low frequency range (9–16 Hz) was observed before and after the task, but visible high frequency activity can only be seen in 15–18 s. The absence of low frequency activity is similar to μ-suppression. The raster plot does not show any distinctive spiking activity correlated with the WEM task. R1's neural activity, shown in Figure 7F, is like that of R2. In L2, Figure 7G, shows activities in both low and high frequency ranges. However, the elevated low frequency activity can only be seen before the task starts. The activities associated with the movement can be seen from 15–100 Hz range in 9–18 s time period. The raster plot shows associated coordinated cross-channel firing in this time period as well. Lastly, in Figure 7H, L1 shows low frequency activity both before and after the task takes place. The strong high frequency activity takes place from 35–100 Hz in 9–18 s time period. The raster plot for L1 shows more activity than any other array and displays the bursts of activity when the task takes place.

#### 3.2.2 Oscillatory activity

**Figure 8:**
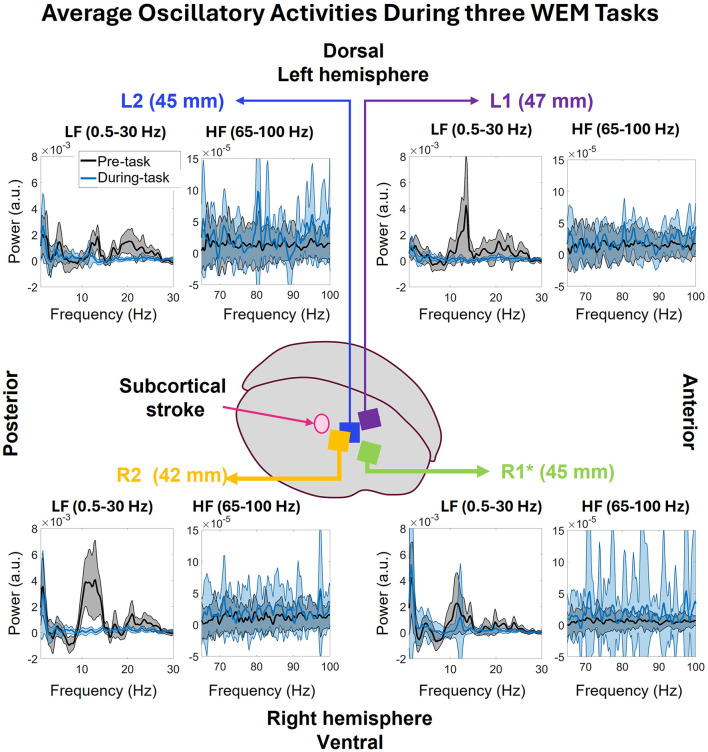
The average oscillatory activities in 0.5–30 and 65–100 Hz bands during the three WEM task divided into pre and during the task. In R2, the low frequency peaks of both pre and during the WEM task are closer in power. Before the task, R2 shows some activities throughout the 0.5–30 Hz band but, during the task, no such activities are observed. In the 65–100 Hz band, average power is higher during the task than before the task. R1 shows activities like R2 before the task in 0.5–30 Hz band. However, during the task, the 1.46 Hz peak has more power than before the task along with a 10–13 Hz peak. In the 65–100 Hz band, the activity is similar to R2. Farther from the stroke, L2 shows stronger activities in 0.5–30 Hz band for pre-task than during the task. On the other hand, the activities in 65–100 Hz band show stronger activity during the task which is expected. L1 shows similar behavior to L2 for across the bands and both conditions except for a peak in 9–15 Hz band before the task in low frequency zone.

The average oscillatory activity of all arrays corresponding to pre- and during- the WEM task is shown in Figure 8 (post-task was equivalent to pre-task so it was not included in the figure). The figure displays power spectrums of the oscillatory activity in low frequency (0.5–30 Hz) and high frequency (65–100 Hz) zones, to observe the frequency domain activity in each zone with the externally cued task, and how the activity differed based on the task state. Starting from closest array to the stroke, R2, in the lower left corner, both before and during the task there are strong peaks in 1.46Hz. Before the task, the average power was 3.54 ± 2.19 × 10^−3^ a.u. and, during the task, the average power decreased to 2.96 ± 3.36 × 10^−3^ a.u. Before the task, strong activity can be seen in 7–16 Hz range along with 16–18 Hz and 18–17 range. However, during the task, there was no more strong activity in R2 in the low frequency range. During the WEM tasks, the average oscillatory activity in the pre-task state is weaker than during-task state which is expected. In R1, (lower right of Figure 8), strong low frequency peaks can be seen at 1.46 both before (4.21 ± 2.74 × 10^−3^ a.u) and during (5.21 ± 8.39 × 10^−3^ a.u) the task. Like R2, other strong activity can be seen in 7–15 Hz range. However, unlike R2, there is a peak in the 10–13 Hz range during the task. In the high frequency range, the activity is much stronger during the task than before the task. In L2 (upper-left of Figure 8), the peaks in the low frequency still happen at 1.46 Hz with 2.00 ± 1.41 × 10^−3^ a.u. before and 2.69 ± 2.49 × 10^−3^ a.u. during the task. The rest of the activity in 0.5–30 Hz range shows strong peaks in 3–9, 9–15, and 15–27Hz ranges. During the task, there are some peaks in 3–6 and 9–14 Hz range. In the high frequency zone, the power is stronger during the task than before the task. The power during the task is comparatively stronger than those seen R1, R2, and L1. There is also a visible peak at 80 Hz. In L1 (upper-left Figure 8), which is the farthest array from the stroke, the low frequency peak before the task happens at 2.20Hz with 1.16 ± 0.74 × 10^−3^ a.u. instead of 1.46 Hz unlike all the other arrays. However, the low frequency peak during the task happens at 1.46 Hz with 1.15 ± 0.75 × 10^−3^ a.u. There is also a strong peak in 9–15 Hz range along with some activities in 15–27 Hz range. In the high frequency zone, the power during the task is stronger than before the task as expected. There is also a peak at 80Hz which is, however, not as strong as the one seen in L2. Among all the correctly implanted arrays, the strongest low frequency peak was seen in R1 and lowest low frequency peak was seen in L1 and both happened before the task.

#### 3.2.3 Oscillatory activity of the entire isometric wrist flexion and extension session

**Figure 9:**
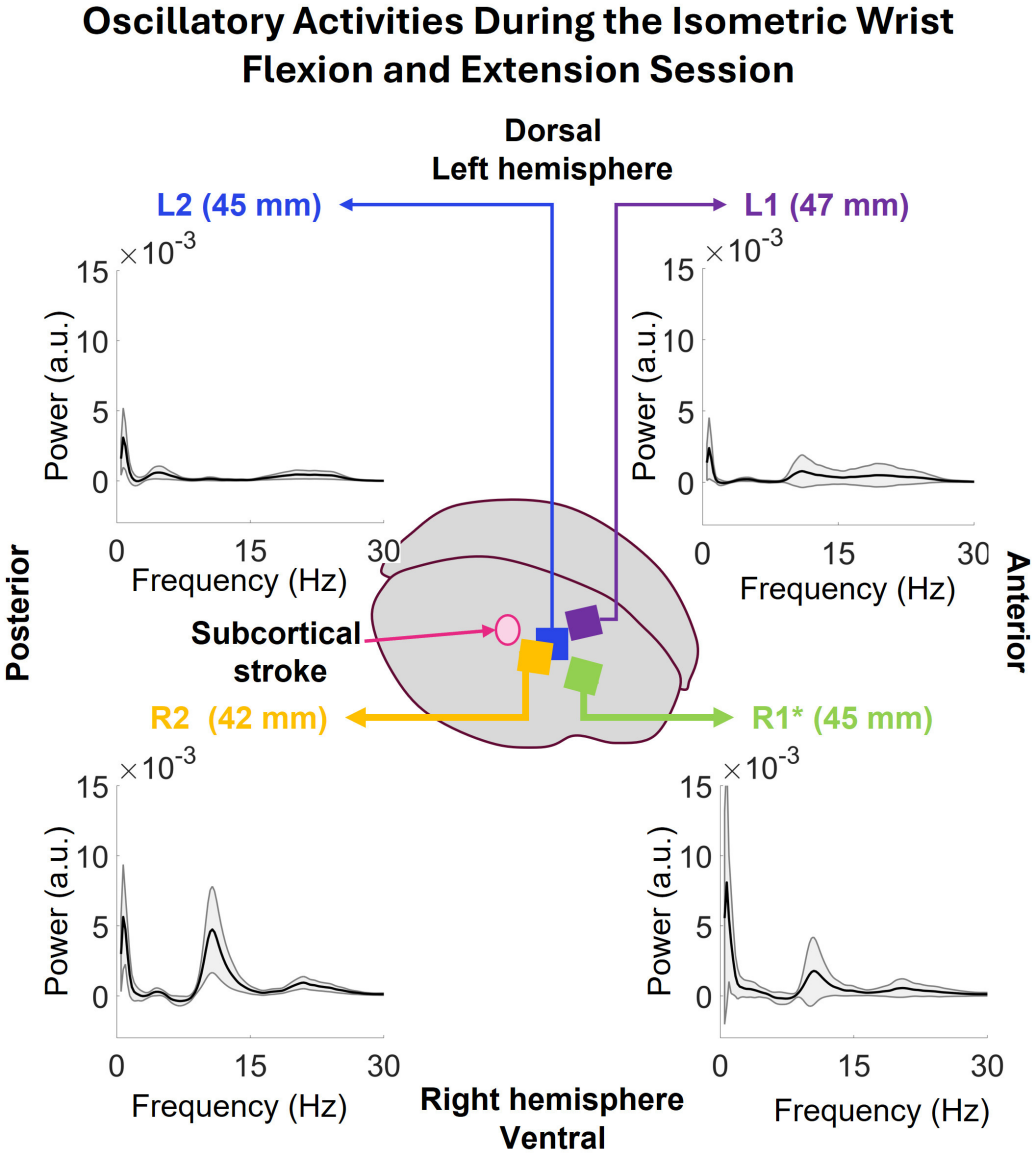
The oscillatory power spectrums of all the arrays during the isometric wrist flexion and extension session. Common low frequency peaks at 0.73Hz are visible in all the arrays. The peaks at R2 and R1 have more power than the ones seen in L2 and L1. Both R2 and R1 have peaks at 8–14 Hz band with some activities in 16–27 Hz. L2 shows activity in 3–8 Hz and 16–27 Hz and L1 shows some activities in 9–28 Hz range.

The oscillatory activity during the full session was also computed for each array, and are presented in Figure 9. The low frequency peaks in all the arrays happen at 0.73 Hz. R2's power at this frequency is 5.63 ± 3.74 × 10^−3^ a.u., R1's 8.09 ± 8.84 × 10^−3^ a.u., L2's 3.08 ± 2.12 × 10^−3^ a.u., and L1's 2.39 ± 2.14 × 10^−3^ a.u. Both R1 and R2 show strong peak in 8–15 Hz range with some activity in 16–27 Hz range. L2 shows some activities in 3–8 Hz and 16–27 Hz ranges which are consistent with the WEM activity. Lastly, L1 shows some activities in 9–27 Hz range.

### 3.3 Analysis of planar reach task data

#### 3.3.1 Population decoding

Table 2 shows the results of population decoding the activity of classified single-unit ensembles relative to the eight cued directions. With the original labels, the Linear SVM scored better than the k-NN model across all metrics and for both arms. The best performance was achieved for the paretic arm—with a 0.4650 accuracy score and 0.3789 F1 score. When the labels were shuffled, the results were not better than chance (12.5%) proving the relationship between the original labels and the neural features. With the randomly selected 100 sets of 20 channels, the overall results were inferior to those obtained with the responsive channels. The results with all 128 channels also show subpar results compared to only using the responsive channels' performance.

**Table 2:** Population decoding of the Kinarm center-out tasks.

**Labels**	**Arm**	**Channels**	**Model**	**Training iterations**	**Average accuracy**	**Average precision**	**Average recall**	**Average specificity**	**Average F1 score**
Original	Unimpaired	19 RC	k-NN	50	0.3740	0.3211	0.3413	0.9106	0.3052
	Paretic	12 RC	k-NN	50	0.3025	0.1941	0.3025	0.9004	0.2254
	Unimpaired	19 RC	Linear SVM	50	**0.3820**	**0.3419**	**0.3688**	**0.9120**	**0.3304**
	Paretic	12 RC	Linear SVM	50	**0.4650**	**0.3415**	**0.4650**	**0.9236**	**0.3789**
Shuffled	Unimpaired	19 RC	k-NN	500	0.1238	0.0810	0.1245	0.8746	0.0902
	Paretic	12 RC	k-NN	500	0.1253	0.0738	0.1253	0.8750	0.4804
	Unimpaired	19 RC	Linear SVM	500	0.1262	0.0818	0.1258	0.8750	0.0909
	Paretic	12 RC	Linear SVM	500	0.1217	0.0716	0.1217	0.8745	0.0848
Original	Unimpaired	20 random (100 sets)	k-NN	50 × 100	0.2432	0.1650	0.2493	0.8918	0.1824
	Paretic	20 random (100 sets)	k-NN	50 × 100	0.2932	0.1797	0.2932	0.8990	0.2100
	Unimpaired	20 random (100 sets)	Linear SVM	50 × 100	0.2670	0.1858	0.2735	0.8952	0.2037
	Paretic	20 random (100 sets)	Linear SVM	50 × 100	0.3238	0.2132	0.3238	0.9034	0.2439
Original	Unimpaired	128	k-NN	50	0.2737	0.2206	0.2738	0.6594	0.2293
	Paretic	128	k-NN	50	0.3382	0.2275	0.3382	0.6435	0.2576
	Unimpaired	128	Linear SVM	50	0.3084	0.2520	0.3260	0.6649	0.2698
	Paretic	128	Linear SVM	50	0.3495	0.2449	0.3495	0.6452	0.2754

The best performances for both the unimpaired and paretic arms are indicated in bold font.

#### 3.3.2 Oscillatory activity of the entire session

**Figure 10:**
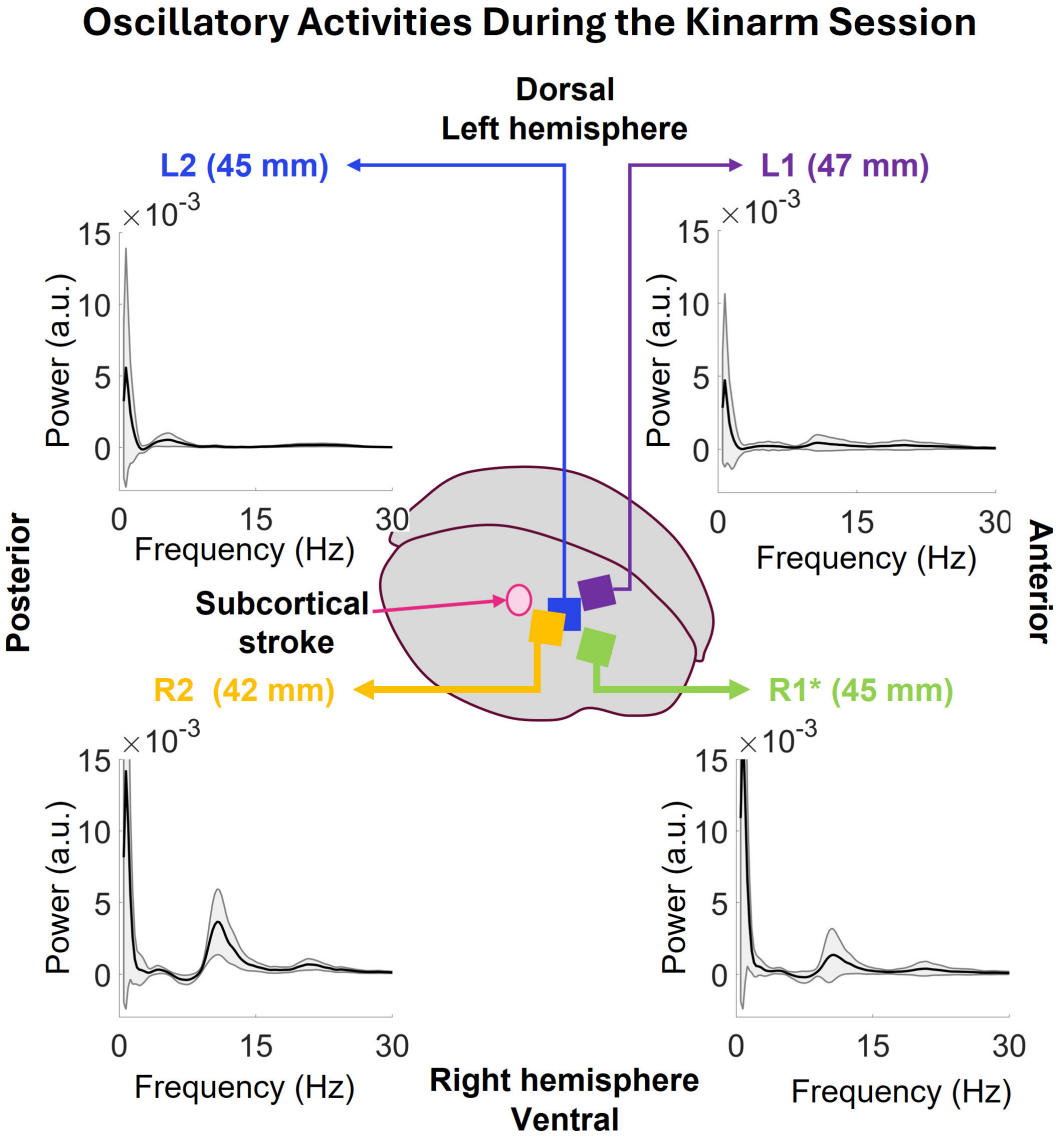
The oscillatory power spectrums of all the arrays during the Kinarm session. Common low frequency peaks at 0.73Hz are visible in all the arrays. The peaks at R2 and R1 have more power than the ones seen in L2 and L1. Additionally, both R2 and R1 have peaks at 7–17 and 16–27Hz bands. L2 shows activity in 2–8 and 16–27 Hz and L1 shows some activity in 8–15 Hz range.

The oscillatory activity during the full Kinarm session was computed for the individual arrays as shown in Figure 10. The low frequency peak in all the arrays happens at 0.73 Hz. R2's power at this frequency is 14.17 ± 16.63 × 10^−3^ a.u., R1's 17.72 ± 20.17 × 10^−3^a.u., L2's 5.57 ± 8.37 × 10^−3^ a.u., and L1's 4.72 ± 5.97 × 10^−3^ a.u. Both R1 and R2 show strong peak in 7–17 Hz range with some activity in 16–27 Hz range which was also seen in the Isometric task session. L2 shows some activities in 2–8 Hz and L1 in 2–27 Hz range.

#### 3.3.3 Oscillatory activity of the Kinarm center-out tasks for both arms

Supplementary Figures S3, S4 show the periodogram, raster plots, and oscillatory activity for unimpaired and paretic arms respectively. In both figures, there are strong low frequency activities in R1 and R2 compared to L1 and L2 with visible peaks in 8–14 Hz range. There were no long coordinated cross-channel firing bursts in these center-out tasks' raster plots as seen in the data from other sessions. Unlike other sessions, where he was primarily performing tasks with his wrist, in this session, he was moving his whole arm.

## 4 Discussion

In this paper we presented an analysis of the intracortical recordings collected from the cerebral cortex in the precentral gyrus overlying a subcortical stroke. Various studies using EEG and magnetoencephalography (MEG) in stroke patients have found the classical delta band (1–4 Hz) increases while alpha, beta, and gamma decrease (Sutcliffe et al., 2022). In our study, among the three correctly implanted arrays, R1 was the closest to the stroke site. This array's power spectrum showed increased power in the low frequency bands, 0.5–4 and 8–30 Hz for all sessions.

It was observed that as the distance between an array and the stroke site increased, the low frequency power decreased, and high frequency power increased. In all sessions, increased low frequency power was evident in R2 (closest to stroke) and attenuated low frequency power evident in L1, the array furthest from the stroke. Elevated high frequency power was observed in the arrays L1 and L2 but attenuated in R2; periodograms of the sessions suggest that high frequency activities correlated with motor movements.

Frequent coordinated cross-channel firing (time-aligned bursts of classified action potentials) was evident during the sessions as shown in Figures 4, 7. Along with these sessions reported in this paper, an additional session's raster plots and the corresponding firing rates shows the same activity in Supplementary Figure S5 along with the MyoPro hand and elbow trajectory. In this case, the data was collected in open-loop while the MyoPro was being operated externally by an investigator. To our knowledge, these coordinated cross-channel firing of action potentials have not been reported before in healthy brain iBCI trials. Interestingly, when the participant moved his whole arm as evident in Supplementary Figures S3, S4 (kinarm center-out for both arms) rather than isolating his wrist, such coordinated cross-channel firing for long duration is not seen.

The cross-channel simultaneous low frequency bursts could reflect a pathologically synchronized network, increased excitability, or disinhibition following stroke. While spectral slowing is ubiquitous in post-stroke EEG (Johnston et al., 2023), in particular overlying the lesion, bursting activity typically is only manifest at the scalp EEG in the context of post-stroke seizures, either as part of an ictal phenomenon or as intermittent interictal aperiodic process (Verma and Kooi, 1986; Saitoh et al., 1992). Aperiodic bursts of epileptiform transients are distinct from periodic lateralized discharges, the latter of which are invariably associated with altered consciousness (Niedzielska et al., 2000). Term neonates with perinatal arterial ischemic stroke who develop seizures exhibit interictal unilateral bursts of theta activity with sharp or spike waves intermixed, and these bursts are distinct from 1–2 Hz seizures (Low et al., 2014). Decreased excitation or enhanced subcortical inhibition has been shown to disrupt the thalamocortical system toward aberrant entrainment manifest as low-frequency bursts (van Wijngaarden et al., 2016). The power spectra of scalp EEG of people with stroke (and without clinical epilepsy) may show a broad reduction in β-band activity, a downward shift of dominant α-peaks, and increased power over the lower frequencies (δ and θ-range). Computational modeling implies that a lesion extending to cortex could lead to sustained cell membrane hyperpolarization in corresponding thalamic relay neurons, leading to de-inactivation of voltage-gated T-type Ca^2+^-channels, the net effect being transition of neurons from tonic spiking to a pathological bursting regime (van Wijngaarden et al., 2016). Once initiated, this thalamic bursting becomes synchronized at a population level via divergent intrathalamic circuits and entrainment of thalamocortical pathways that propagate the low frequency bursts beyond the lesion location. A resting state magnetoencephalography study with chronic stroke patients found that abnormally elevated low frequency power was best explained by a steepening of the aperiodic component of the power spectrum, rather than an enhancement of low frequency oscillations (Johnston et al., 2023). A large-scale model of the neocortex implied that post-stroke changes in excitatory-inhibitory homeostasis drove widespread increases in excitability (dos Santos et al., 2023). EEG spectral slowing, and changes in the power-law decay, when quantified as a spectral exponent metric, reliably correlates with clinical recovery (Lanzone et al., 2022).

Our results confirm the widely observed spectral slowing seen in scalp EEG (increased low frequency and attenuated high frequency spectral power) at a higher spatial resolution in an intracortical ipsilesional location, and reveal cross-channel bursts of action potentials. In the context of prior literature and modeling, the spectral slowing may result from the disrupted linkage between the recorded cortex and the thalamus disrupting excitation-inhibition homeostasis and unmasking an aberrant entrainment and pathological bursting arising from spared thalamus propagating into ipsilesional cortex. Computational modeling might help clarify if the cross-channel bursts of action potentials that we observe are more consistent with increased low frequency oscillations, or if instead they represent single unit ensemble autocorrelations in the time domain that are steepening the aperiodic components independently of low frequency oscillations. These findings bear upon future neuro restorative strategies: first, our results suggest that usable voluntary motor control signals can be derived in spite of the evident post-stroke excitation-inhibition homeostasis and thalamocortical activity disruptions; second, neuromodulation strategies (vagus nerve stimulation, spinal cord stimulation, non-ablative functional ultrasound, cortical stimulation) that normalize the spectral slowing and action potential hypersynchrony, could improve the yield of BCI decoders recording cortical activity.

This study presented three offline features: average firing rates, average power spectral density (PSD), and average phase-amplitude coupling (PAC), which displayed modulation with the closing movements initiated by the participant. The extraction of PAC using the modulation index (MI) (Tort et al., 2010) is a computationally demanding process and may not be trivially implemented for real-time applications. However, real-time implementations of PAC and the estimation of phase and amplitude have been proposed, which can be adapted for online PAC feature extraction (Dellavale et al., 2013; Lu et al., 2018; Rosenblum et al., 2021; Salimpour et al., 2022). Lu et al. (2018) introduced MSPACMan (Multirate Sub-banded Phase-Amplitude Coupling for Microelectrode Acquisitions with Noise), a Python library that computes MI using vectorized calculations with popular Python libraries such as NumPy and SciPy. Their optimization methodology introduced filtering in the frequency domain rather than the time domain, which allowed them to apply FFT only once per data segment. The vectorization process, combined with parallel processing, significantly reduced the runtime. MSPACMan required only 0.1 s to calculate 225 PAC values compared to 33.8 s with the traditional implementation. The results demonstrated that this method yielded PAC values similar to the traditional implementation, even with a low signal-to-noise ratio, where the PAC value increased as the noise decreased, and vice versa. Due to its faster computation time and robustness to noise levels, MSPACMan can be considered for real-time PAC computation in future work.

The population decoding performance of the planar reach task was not as high as that of similar studies. Using 12 responsive channels' leaky integrated firing rate features, we achieved 46.5% accuracy for the paretic arm. In a study establishing an animal model for iBCI, a monkey implanted with a 10 × 10 microelectrode array performed a four-directional center-out task using its wrist. The firing rates showed highly discriminatory properties (105/149 neurons), and the four directions were classified with a 96% accuracy using an SVM (Zhang et al., 2012). In a human study evaluating intra-day signal stabilities, two participants with ALS achieved 56±22% in a four-directional and 70±22% in an eight-directional center-out task (Perge et al., 2014). A more recent human study implemented a decoder based on a recurrent neural network for eight-directional center-out cursor control and tested it in real-time where the decoder performed with an average of 93.8% accuracy in this time over 3 months (Hosman et al., 2023). Since the participants of these two studies were tetraplegic with intact cerebrums, the tasks were imagined rather than physically performed, as in the Cortimo trial. Nevertheless, despite the Cortimo participant being stroke-affected, we successfully identified discriminatory features and performed population decoding.

This study is limited by several factors. First, the data was collected from a single participant, rendering it impossible to determine which neurophysiological patterns might be representative of the stroke-affected cerebral cortex rather than idiosyncratic features of this one individual. For example, the participant had fluctuating arousal: sometimes he was alert and intensely engaged, and other times he would become distracted and fall asleep. Second, HD-sEMG and Kinarm data were only recorded on a single day, precluding inference of longitudinal patterns. Third, the participant was ambulatory and could move the unimpaired limbs of his body as he wished, which could have added noise to the neural data collected.

In most previously published results of iBCI trials, the neural machinery of motor control in the brain was intact up until the area of injury (e.g., infarction of the pyramidal tracts due to basilar artery occlusion, or traumatic injury of the cervical spinal cord). When stroke affects one or more other regions of the brain beyond the brainstem, more aspects of motor control and other systems can be disrupted. Our experience of the n-of-1 trial highlighted several challenges that may need to be considered when developing iBCIs for people with cerebral stroke. While we do not propose that the deficits seen in our one participant are necessarily generalizable across all individuals with cerebral stroke, we anticipate that some of these challenges would occur in other individuals. These challenges included:

*Inconsistent task performance*. Our participant exhibited behavioral processing delays in performing tasks with his unimpaired upper extremity, suggesting a central cognitive deficit. Such processing delays could arise from disruptions in mood, memory, cognition, visual perception, processing speed, executive control, and proprioception due to direct damage to corticofugal fibers. It is possible that repeatable patterns of activity could be present in the cortical areas overlying chronic stroke, yet the consistency of their timing could be altered. If repeatable patterns of activity were present, conceivably they could be modeled on a per patient basis to optimize the decoder performance. A sliding window search algorithm and time-warping techniques (Willett et al., 2021) could be beneficial in iBCI users with history of intracerebral stroke.

*Gradient of intact and impaired function and unintended motor activation*. Our participant exhibited major impairments in finger and wrist extension but retained some degree of finger and wrist flexion. If asked to imagine hand-open-and-close, the participant would have to suppress actually opening the hand. When instructed to extend the fingers and wrist, for example, he would inadvertently activate finger and wrist flexors. Hence, instructions to imagine or attempt to perform a hand opening-and-closing sequence are inherently more confusing than for a person who has no impairment or has complete motor disconnection. Even if these unintended movements arose subcortically (e.g., unopposed reticulospinal or rubrospinal output), once engaged, they would mechanically interfere with the resulting movement and cause proprioceptive feedback that could in turn manifest in the cerebral cortex, contaminating the neural features used for an iBCI decoder.

*Mechanical and spasticity effects on motor decoding*. Our participant exhibited a combination of spasticity (velocity-dependent mechanical resistance) and mechanical changes (shortening of flexor tendons) in his upper extremity, which caused mechanical opposition to the intended, triggered orthosis motion. This mechanical opposition provided sensory feedback that may have in turn triggered further aberrant motor instructions in an attempt to overcome the opposition.

## 5 Conclusion

Intracortical brain-computer interfaces comprise a class of medical devices intended to restore function in people with neurological disease and injury. While these technologies have been evaluated in individuals with locked-in syndrome due to brainstem stroke, that type of stroke syndrome fortunately is rare. Most strokes are not confined to focal lesions of the brainstem, and often involve the cerebral hemispheres. Strokes can be classified by the areas injured (e.g., cortical or subcortical, basal ganglia, thalamus, etc.), the mechanism (ischemic or hemorrhagic), and etiology (e.g., cardioembolic, AVM rupture, large vessel disease, etc.). Given that stroke is a common etiology of chronic disability, understanding how iBCIs perform in more common types of stroke is important to ensure such devices benefit that larger population. By analyzing data recorded from one individual with chronic subcortical stroke (embolic stroke of unknown source), we discovered activity patterns that had not been reported in other iBCI trials. Although one cannot disambiguate whether these patterns are generalizable across other types of cerebral stroke, the power spectral features observed mirror EEG and MEG findings in the stroke population and highlight the possibility that the feature selection and decoder approach used may require unique considerations to build iBCI devices that can help this population. Our findings suggest that it may be advantageous to consider the anatomical location of implanted sensors relative to the stroke: iBCI technologies that either cover a large surface area or are purposefully positioned far enough away from the center of the stroke, yet still within areas known to be easily brought under voluntary modulation, could spell the difference between the device being effective or not. In our n-of-1 trial, the high gamma spectral power recorded by intracortical arrays was used to drive real-time control of a powered upper extremity orthosis. The results suggest that various features of the neural activity recorded from the ipsilesional precentral gyrus can be used to decode intended hand aperture and overall hand direction. Our offline analyses presented in this paper imply that useful input features for future iBCIs may include phase-amplitude coupling and the firing rates of single units found to have a high modulation index.

## Data Availability

The data analyzed in this study is subject to the following licenses/restrictions. The data will be made available to researchers who provide a methodologically sound proposal to achieve the aims of the approved proposal. To gain access, data requestors will need to sign a data access agreement. Requests to access these datasets should be directed to mijail.serruya@jefferson.edu.
