# Supplementary material for: Neural signal analysis in chronic stroke: advancing intracortical brain-computer interface design
Source: Front Hum Neurosci. 2025 Feb 21;19:1544397. doi: 10.3389/fnhum.2025.1544397 (PMC11885313; doi:10.3389/fnhum.2025.1544397)
Supplement: Supplementary file 1 [file Data_Sheet_1.pdf]

# *Supplementary Material*

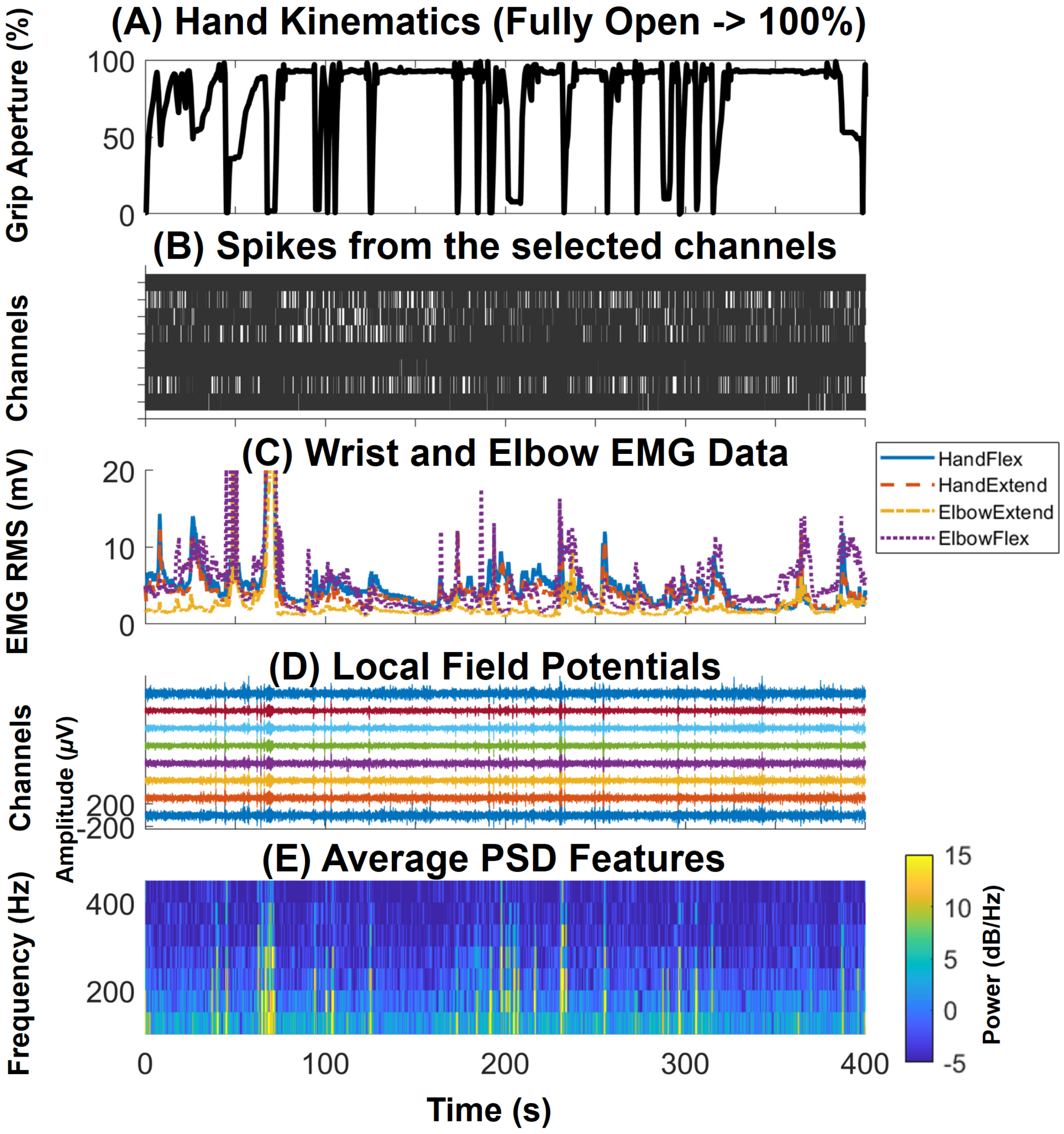


**Figure S1.** Neural signals recorded during the ARAT iBCI task, along with calculated features from local field potentials (LFPs) in real-time **(A)** The grip aperture of the hand demonstrating opening and closing is shown for 400 seconds. **(B)** The spike raster plot of the eight most neuromodulated channels. **(C)** The rectified EMG RMS (mV) of MyoPro from the wrist flexors, wrist extensors, biceps, and triceps. The participant was moving his elbow while the iBCI controlled his hand. **(D)** The LFP traces of eight channels show activities during the closing movements. **(E)** The average periodograms of the eight channels show high frequency activity in the 100-500 Hz range that triggered the closing during the real-time session.

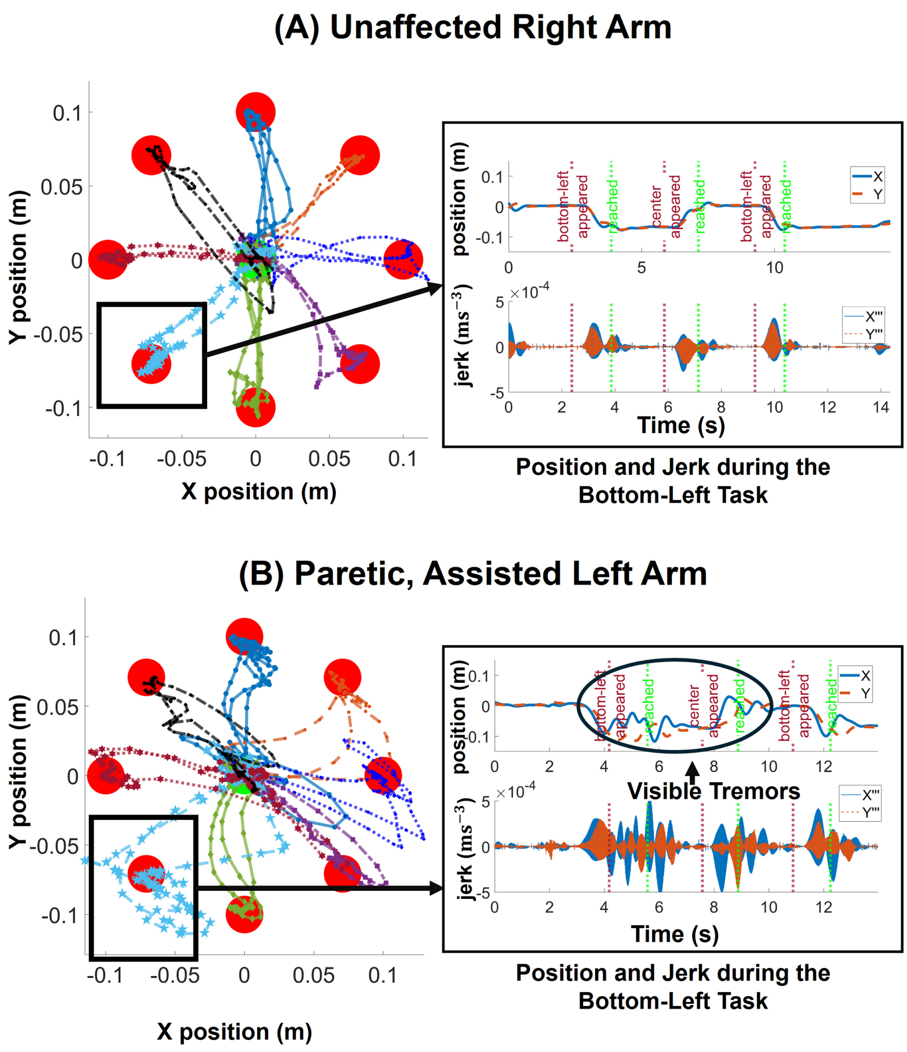


**Figure S2.** The Kinarm center-out tasks performed with both arm shows the difference between the movement of the unaffected right arm and paretic left arm. At the beginning of each trial, the center target would appear red and the participant was instructed to move the cursor over the target and rest there for two seconds, at which time target would become green. After the center target turned green, one of the circumferential targets would turn red, and the center target would disappear. The participant then needed to reach the target to make it turn green. After two seconds, the directional target disappeared, the green center target reappeared and became red. In the given trial time of 14 seconds, the participant needed to toggle between the center and the current directional target as many times as possible. After a break, a new trial begins with another different peripheral target. **(A)** The kinematic trajectories of the right arm toward the center-out and out-to-center targets using the unaffected right arm. The inset shows the position and jerk during the bottom-left target which shows smooth movement. The participant reached the bottom-left target twice and center target once within the allotted 14 seconds. **(B)** The kinematic trajectories of the left arm (assisted by the right arm) show more erratic movement. In the inset, the position and jerk show tremors as the participant toggled between the center and bottom-left targets during the 14-second trial time.

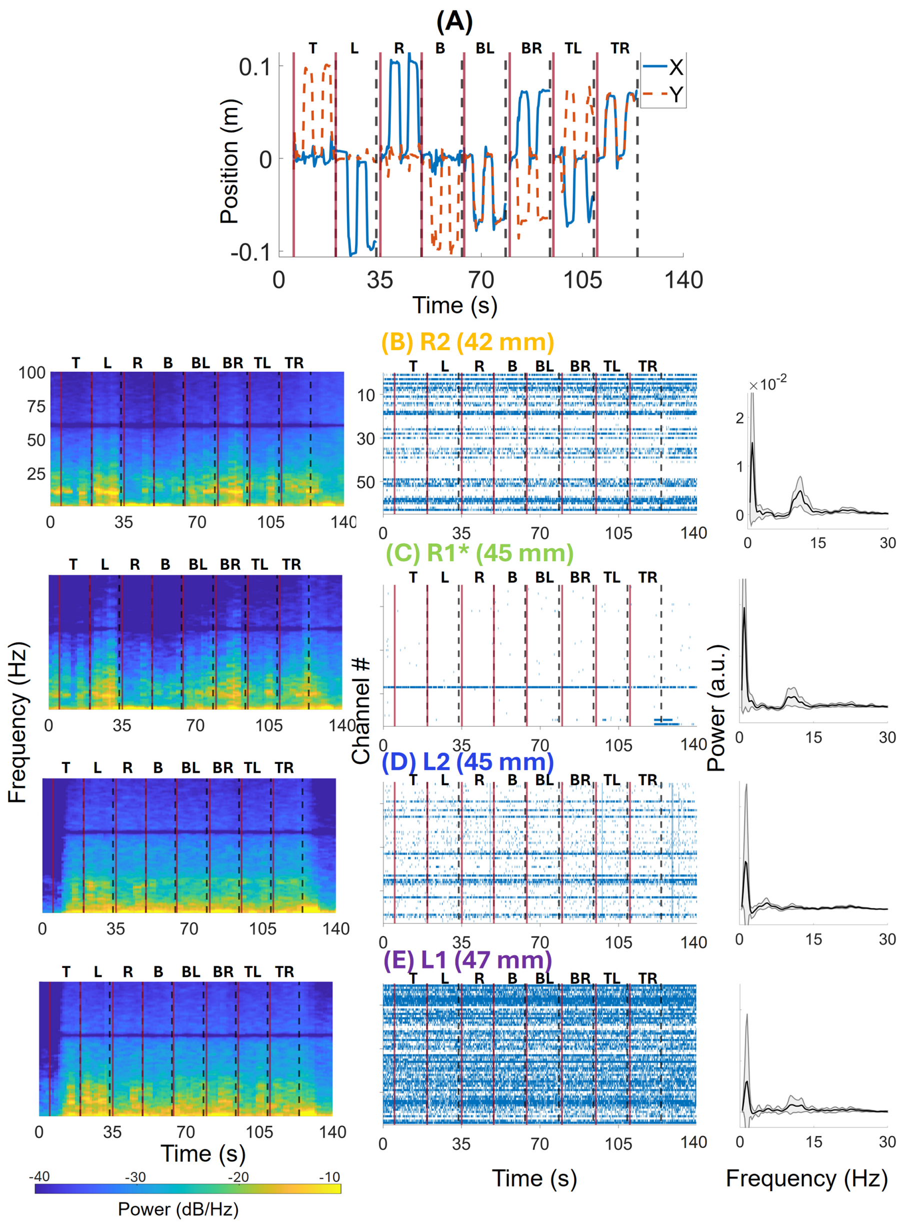


**Figure S3.** The periodograms and the oscillatory activities during the kinarm center-out task performed with the unimpaired right arm. **(A)** The position of the manipulandum in X and Y direction with time. The dark red vertical solid lines show the start of a trial and the black vertical dashed lines show the end of a trial. The directions are shown in abbreviated form on top of each trial. **(B)** The periodogram of 0.5 -100Hz averaged over all the channels of the R2 shows low frequency activities for all during directions except for right (R) and bottom (B). In R2 and R1. The raster plot lack coordinated cross-channel firing. The oscillatory activity calculated for the 140-second of data corresponding to this center-out task show peaks in 0.5-2Hz and 9-14 Hz range that can be associated with low-frequency activities observed in the periodogram. **(C)** R1 shows similar activities in the periodogram, raster plot, and oscillatory plot. The high frequency activities are weaker than R2 in this array. **(D)** In L2, the low and high frequency activities is negligible before the first trial and after the last trial. The low frequency activities do not show any distinct pattern which is also evident in the oscillatory plots. The rater plot lacks long duration of coordinated cross-channel firing which might be the result of moving the whole arm, not just the wrist. **(E)** L1 shows similar activity in both to L2 before and after the task in the full 0.5-100Hz range. The low frequency activities in 8-14Hz band are visible for all directions which can also be seen in oscillatory plot. The raster plot also lacks coordinated cross-channel firing like L2.

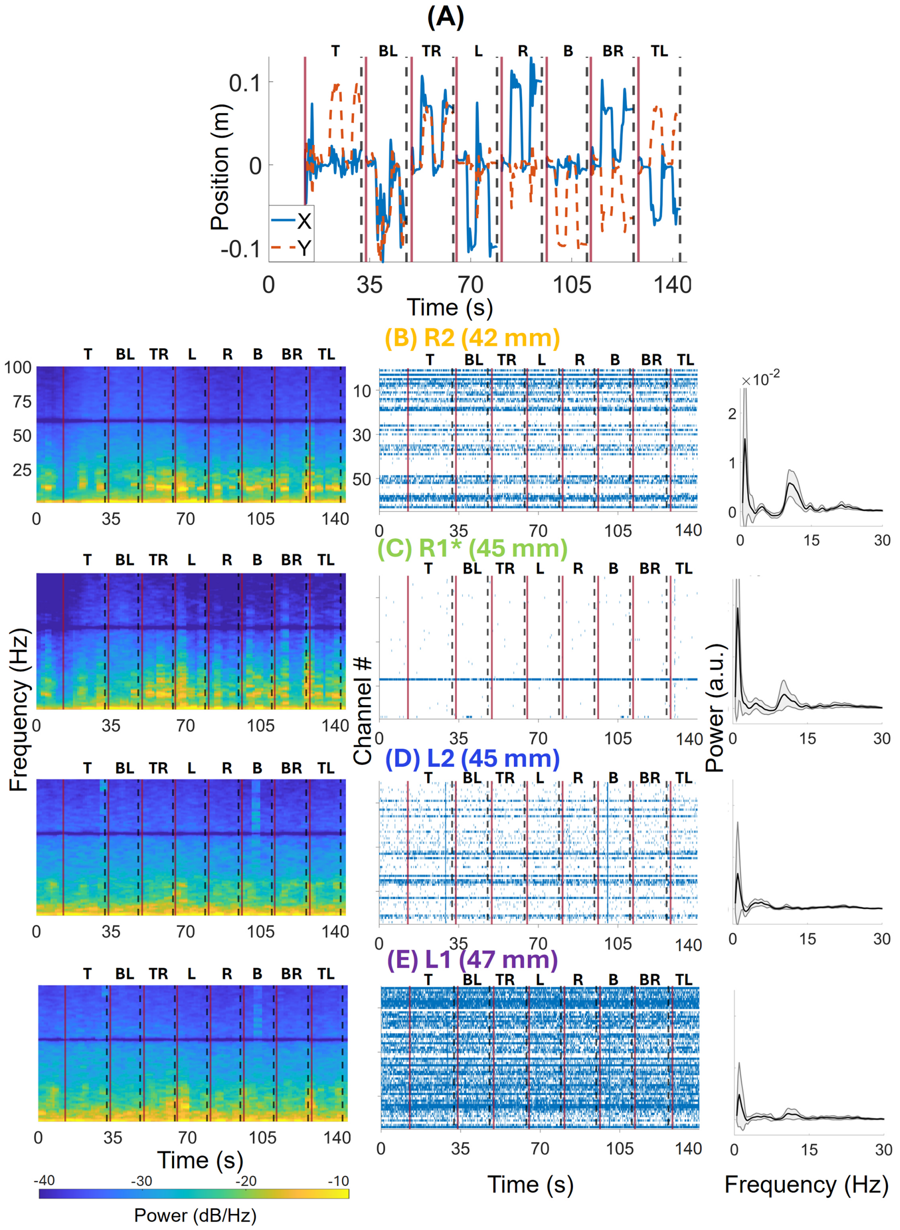


**Figure S4.** The periodograms and the oscillatory activities during the kinarm center-out task performed with the paretic left arm. **(A)** The position of the manipulandum in X and Y direction with time. The dark red vertical solid lines show the start of a trial and the black vertical dashed lines show the end of a trial. The directions are shown in abbreviated form on top of each trial. The movements are less smooth than those performed by the unimpaired arm. **(B)** The periodogram of 0.5 -100Hz averaged over all the channels of the R2 shows low frequency activities for all during directions except for right (R) and bottom (B). The raster plot lacks coordinated cross-channel firing. The oscillatory activity calculated for the 140-second of data corresponding to this center-out task show peaks in 0.5-2Hz and 9-14 Hz range that can be associated with low-frequency activities observed in the periodogram. **(C)** R1 shows similar activities in the periodogram, raster plot, and oscillatory plot. The high frequency activities are weaker than R2 in this array. **(D)** In L2, the low and high frequency activities are similar throughout the trials. The low frequency activities do not show any distinct activity which is also evident in the oscillatory plots. The raster plot lacks long duration of cross-channel firing which might be the result of moving the whole arm, not just the wrist. However, there are two short duration cross-channel firing that happen around the same times of two broad-brand activities at 30 and 100 seconds. **(E)** L1 shows similar activities like L2 in the full 0.5-100Hz range for the whole duration of the trial. The low frequency activities in 8-14Hz band are visible for all directions which can also be seen in oscillatory plot. The raster plot also lacks cross-channel firing like L2 but shows similar cross-channel activities around 30 and 100 seconds.

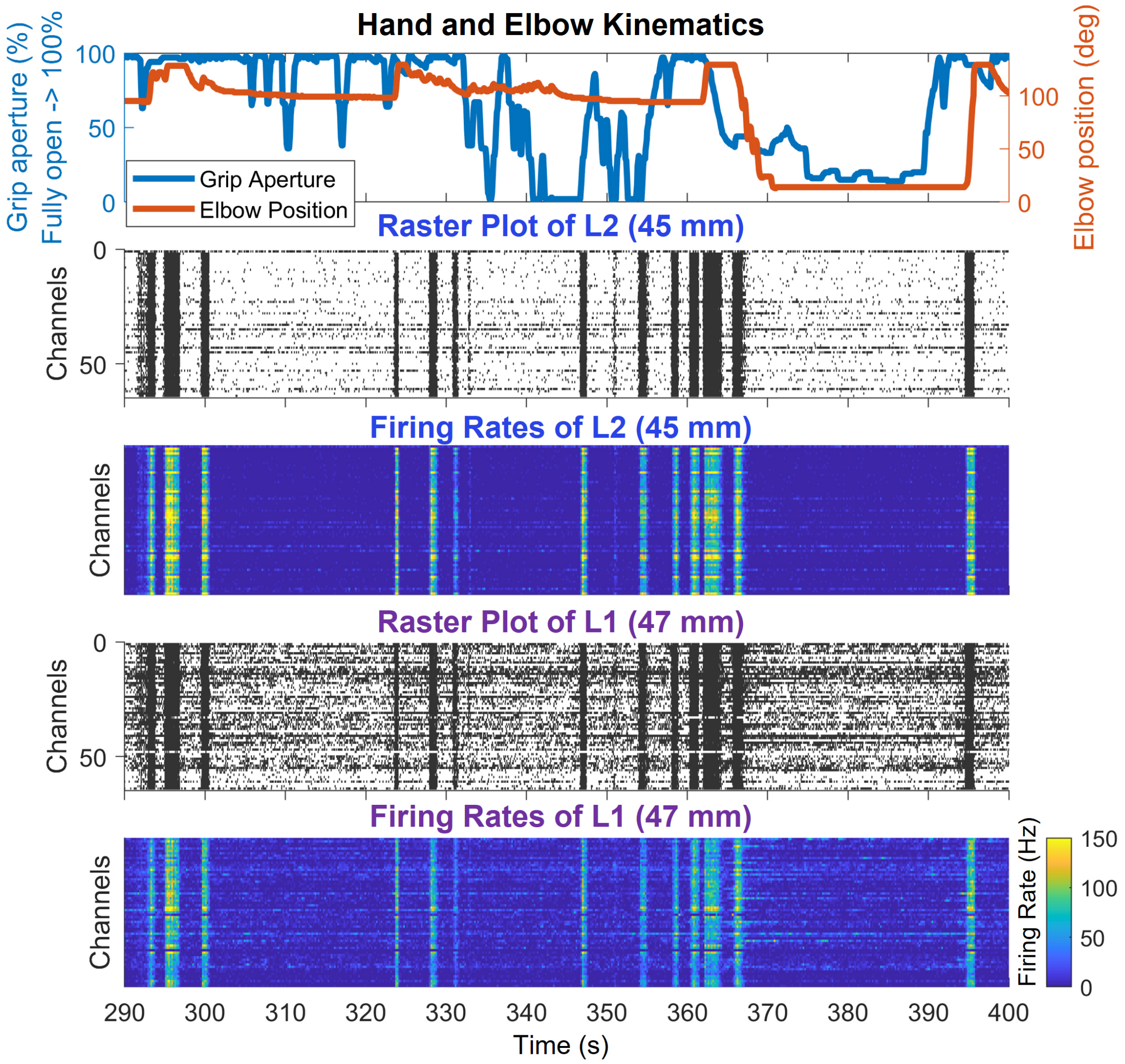


**Figure S5.** The frequent coordinated cross-channel firing in a stroke brain happening across the majority of channels are not common in a healthy brain. The firing rates were calculated with a 200ms bin without overlap. This data was collected in a session where the MyoPro was externally controlled by an expert, not the participant.
